# Mesenchymal Stem Cell‐Derived Extracellular Vesicles Modulate the Course of Peritoneal Inflammation Through Metabolic and Epigenetic Regulation

**DOI:** 10.1002/advs.202508645

**Published:** 2025-11-26

**Authors:** Qiang Huang, Yuxiang Sun, Pengpeng Kuang, Juan Sun, Dandan Guo, Long Peng, Hu Zhou, Qinrong Song, Zhihao Huo, Canming Li, Janusz Witowski, Zhaoyong Hu, Hui Peng

**Affiliations:** ^1^ Nephrology Division Department of Medicine The Third Affiliated Hospital Sun Yat‐sen University Guangzhou 510630 China; ^2^ Guangdong Lung Cancer Institute Guangdong Provincial Key Laboratory of Translational Medicine in Lung Cancer Guangdong Provincial People's Hospital Guangdong Academy of Medical Sciences Southern Medical University Guangzhou 510080 China; ^3^ Division of Cardiovascular Medicine Department of Medicine The Third Affiliated Hospital Sun Yat‐sen University Guangzhou 510630 China; ^4^ Department of Pathophysiology Poznan University of Medical Sciences Poznan 61‐701 Poland; ^5^ Nephrology Division Department of Medicine Baylor College of Medicine Houston 77030 USA

**Keywords:** CCL2, extracellular vesicles, histone lactylation, mesenchymal stem cell, peritoneal inflammation

## Abstract

Peritoneal dialysis (PD), as a renal replacement therapy, relies heavily on the structural and functional integrity of the peritoneum. In some patients, however, the peritoneum may undergo adverse remodeling and fibrotic thickening, resulting in treatment failure. Here, a previously unrecognized metabolic‐epigenetic mechanism contributing to peritoneal fibrogenesis is uncovered, wherein lactate accumulation in injured peritoneal mesothelial cells promotes histone H3K18 lactylation and transcriptional activation of macrophage‐recruiting chemokine CCL2. In a mouse model of peritoneal fibrosis induced by chlorhexidine gluconate (CG) or PD fluid, the administration of extracellular vesicles derived from human bone marrow mesenchymal stem cells (MSC‐EVs) significantly ameliorates histological and functional changes in the peritoneum. Single‐cell RNA sequencing reveals that MSC‐EVs attenuate mesothelial‐macrophage crosstalk by suppressing CCL2 signaling. Mechanistically, MSC‐EVs reprogram glycolytic metabolism in mesothelial cells, reduce lactate production, and inhibit H3K18 lactylation‐dependent transcriptional activation of CCL2. Pharmacologic blockade of lactate production recapitulates the protective effects of MSC‐EVs. These findings suggest that lactate‐induced histone lactylation is a key driver of peritoneal fibrosis, positioning MSC‐EVs as a promising cell‐free therapeutic strategy for targeting metabolic‐epigenetic inflammation in serosal injury.

## Graphical abstract

MSC‐EVs alleviate peritoneal inflammation and fibrogenesis by disrupting the H3K18la‐CCL2 axis and crosstalk between mesothelial cells and Ly6c2⁺ macrophages. Model cartoons are constructed using the Biorender website (https://www.biorender.com/), and a usage license is obtained.

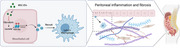

## 1 Introduction

Peritoneal fibrosis represents adverse tissue remodeling that may complicate long‐term peritoneal dialysis (PD), recurrent peritonitis episodes, or neoplastic peritoneal dissemination.^[^ ^1^ ^]^ When occurring during PD, this may lead to peritoneal membrane dysfunction and treatment failure.^[^ ^2^, ^3^ ^]^ Histopathological hallmarks of peritoneal fibrosis include leukocyte‐rich inflammatory infiltration, mesothelial‐to‐mesenchymal transition (MMT), fibroblast activation and expansion, as well as neovascularization and vasculopathy.^[^ ^4^, ^5^, ^6^ ^]^ Currently, apart from the prevention of bacterial peritonitis and the use of biocompatible peritoneal dialysis fluids, there is no widely available treatment option aimed explicitly at preventing peritoneal fibrosis during peritoneal dialysis.

Mesenchymal stem cell (MSC)‐based therapies have revolutionized regenerative medicine, particularly in the context of fibrotic diseases.^[^ ^7^, ^8^ ^]^ MSCs possess several therapeutic advantages, including the potential for multilineage differentiation, immune tolerance, and the ability to home to sites of tissue injury.^[^ ^9^ ^]^ Recent studies have shown that MSC‐derived extracellular vesicles (MSC‐EVs) can act as paracrine mediators, delivering bioactive cargoes to recipient cells while avoiding the risks associated with cell transplantation.^[^ ^10^, ^11^, ^12^ ^]^ In this respect, it has been reported that murine bone marrow MSC‐derived exosomes can ameliorate peritoneal fibrosis in animal models.^[^ ^13^ ^]^ However, it is unclear whether extracellular vesicles derived from human MSCs can exert similar protective effects. Moreover, the underlying mechanisms through which these vesicles execute their activities remain to be fully elucidated.

Recent studies have established cellular metabolic reprogramming as a hallmark of fibrotic progression.^[^ ^14^, ^15^, ^16^ ^]^ Our previous study involving metabolomic profiling has demonstrated that exposure to glucose‐based peritoneal dialysis fluids can trigger a Warburg‐like shift in peritoneal mesothelial cells, leading to increased glycolytic flux and lactate overproduction. Such a metabolic alteration provides both energy and biosynthetic precursors for the synthesis and accumulation of the extracellular matrix (ECM). Importantly, inhibition of glycolytic enzymes has been shown to alleviate peritoneal fibrosis in preclinical models.^[^ ^17^ ^]^ In parallel, increasing evidence suggests that specific metabolites can directly influence chromatin structure through epigenetic modifications.^[^ ^18^, ^19^ ^]^ Histone lactylation, a newly discovered post‐translational modification by glycolysis‐generated lactate, is of particular interest as it has been shown to regulate macrophage polarization^[^ ^20^ ^]^ and gene expression in neuroinflammation and cancer.^[^ ^21^, ^22^, ^23^ ^]^ Histone lactylation has also been implicated in hepatic fibrosis by promoting the activation of hepatic stellate cells.^[^ ^24^ ^]^ It is not known, however, whether similar effects of lactate occur in the peritoneum and contribute to peritoneal fibrosis during PD.

In this study, we investigated the therapeutic effects of extracellular vesicles derived from human bone marrow MSCs in the murine model of chlorhexidine gluconate (CG) or PD fluid‐induced peritoneal fibrosis. By integrating single‐cell transcriptomics, metabolic profiling, and epigenetic analyses, we investigated how MSC‐EVs modulate the peritoneal microenvironment and disrupt profibrotic signaling pathways. Particular attention was given to the interplay between glycolytic metabolism, lactate production, and histone modifications in mesothelial cells. Our findings reveal an unexpected connection between metabolic rewiring and epigenetic regulation in peritoneal fibrogenesis, pointing toward a previously unrecognized mechanism of MSC‐EV‐mediated protection.

## 2 Results

### 2.1 Purification and Characterization of MSC‐EVs

**Figure 1:**
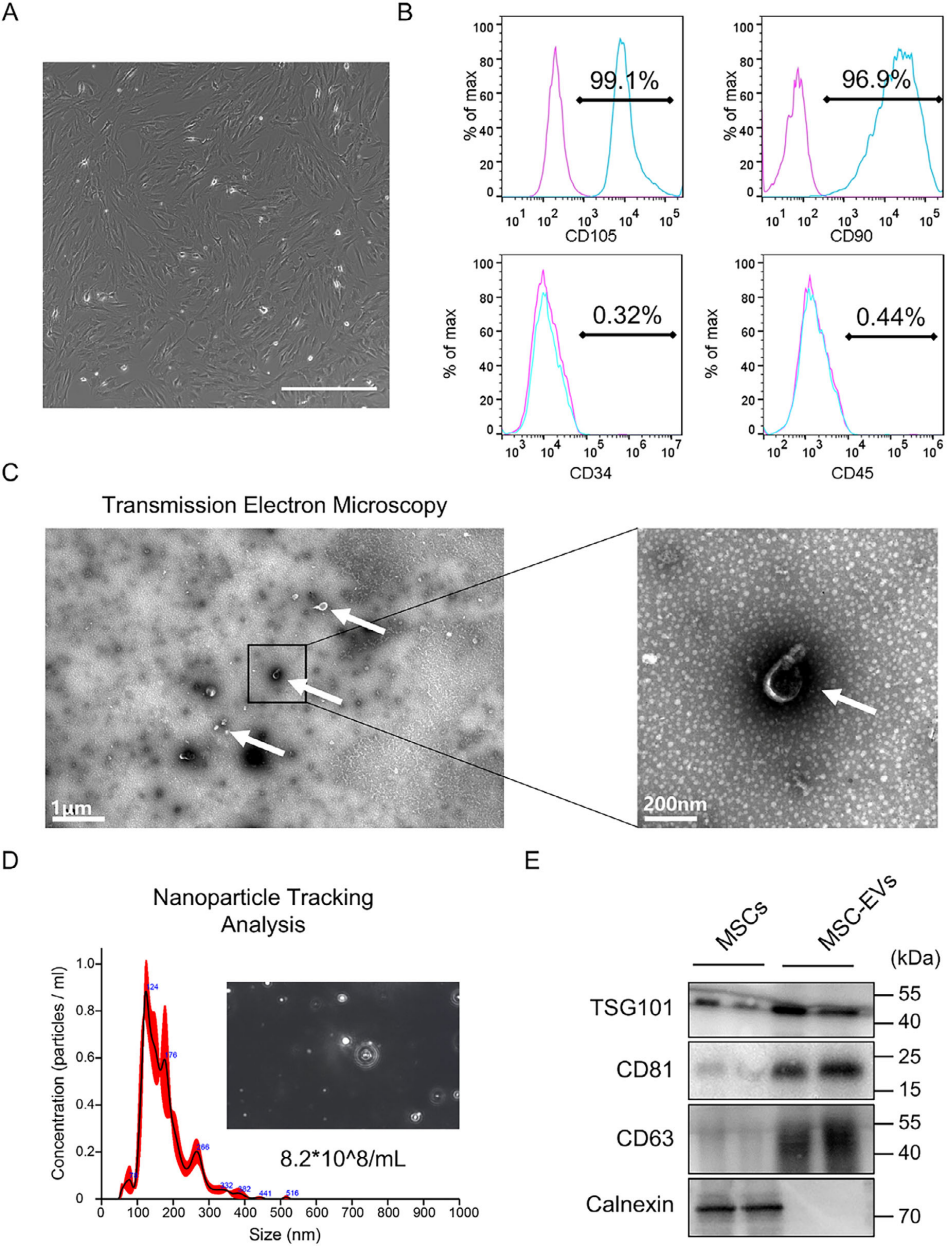
Characterization of MSC‐EVs. A) Optical microscopy revealed that MSCs exhibited a favorable growth morphology. Scale bar, 100 µm. B) Phenotypic marker analysis of MSCs. Sixth‐passage MSCs were stained either with test antibodies (blue) or control antibodies (pink) and analyzed by flow cytometry. Values indicate the proportion of positively stained cell populations. C) Representative electron micrographs of nanoscale vesicles. Left panel: low‐magnification field, scale bar: 1 µm; right panel: high‐magnification field, scale bar: 200 nm. D) Nanoparticle tracking analysis demonstrating the particle concentration and diameter distribution of these EVs. E) Western blot analysis of classical EV markers (CD63, CD81, and TSG101) in the purified EVs.

We isolated extracellular vesicles (EVs) from the conditioned medium of human bone marrow‐derived mesenchymal stem cells (MSCs). Before EV collection, phase‐contrast microscopy confirmed that cultured MSCs exhibited typical spindle‐shaped morphology and robust adherence under standard in vitro conditions (**Figure** **1**A). Flow cytometric analysis demonstrated that the MSC population was uniformly positive for MSC surface markers CD105 and CD90 (≥95%), and negative for hematopoietic markers CD45 and CD34 (≤2%), confirming their phenotypic identity (Figure 1B).

EVs were purified using differential ultracentrifugation and subsequently visualized by transmission electron microscopy (TEM), which revealed spherical, lipid‐bilayered vesicles consistent with exosomal morphology (Figure 1C). Nanoparticle tracking analysis (NTA) showed that the majority of particles ranged from 100–200 nm in diameter, with a peak modal size of ≈124 nm (Figure 1D), consistent with the expected size distribution of small EVs. Western blot analysis further validated EV identity by detecting the presence of classical EV markers CD63, CD81, and TSG101, while confirming the absence of Calnexin, an endoplasmic reticulum marker, thereby excluding significant cellular contamination (Figure 1E).

Collectively, these results confirmed the successful isolation and biochemical characterization of MSC‐derived EVs.

### 2.2 Cell‐Free MSC Therapy Reverses Structural and Functional Deterioration of the Peritoneum

**Figure 2:**
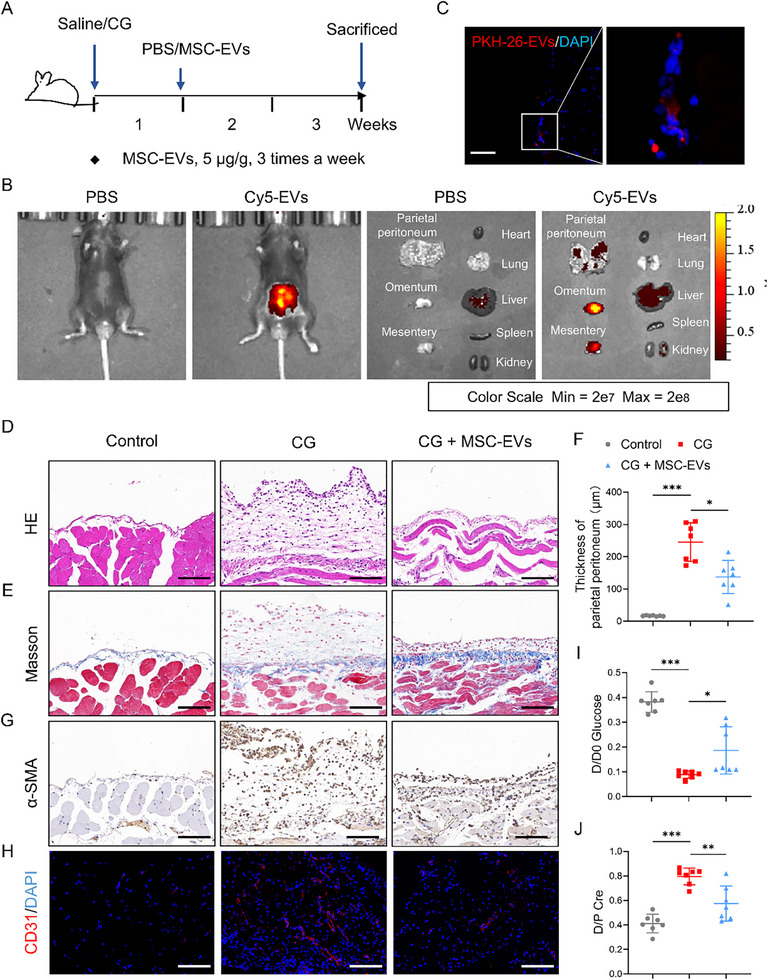
Cell‐free MSC therapy reverses structural and functional deterioration of the peritoneum. A) An overview of the experimental design. Mice received intraperitoneal injections of 0.1% CG or saline three times per week for 3 weeks. MSC‐EVs (5 µg g^−1^ body weight) or PBS were administered intraperitoneally starting on day 7. B) Ex vivo imaging of the main organs from CG model mice, after intraperitoneal injection with Cy5‐labeled EVs or an equivalent volume of PBS for 24 h (n = 3). C) Representative fluorescence images showing uptake of PKH26‐labeled MSC‐EVs (red) by peritoneal cells in CG‐treated mice. Nuclei were counterstained with DAPI (blue) (n = 5). Scale bar, 50 µm. D,E) Representative histological images of peritoneal tissue sections from each group: (D) hematoxylin and eosin (H&E) staining; (E) Masson's trichrome staining highlighting collagen deposition (n = 7). Scale bars, 50 µm. F) Quantification of peritoneal thickness in each group (n = 7). G,H) Representative immunohistochemical staining of fibrotic and angiogenic markers: (G) α‐SMA and (H) CD31 (n = 7). Scale bars, 50 µm (G) and 100 µm (H). I,J) Modified peritoneal equilibration test assessing membrane transport function: (I) D/D0 glucose ratio and (J) D/P creatinine ratio (n = 7). Data are presented as mean ± SD. ^*^ *p* <0.05, ^**^ *p* <0.01, ^***^ *p* <0.001 by one‐way ANOVA.

To evaluate the therapeutic efficacy of MSC‐EVs in vivo, we established a murine model of peritoneal fibrosis induced by intraperitoneal injection of 0.1% CG three times per week for 3 weeks (**Figure** **2**A). Starting on day 7 after the initial CG injection, mice were treated with either MSC‐EVs (5 µg g^−1^ body weight) or PBS control, administered intraperitoneally at the same frequency until the study endpoint (n = 7 per group).

To investigate the in vivo distribution and tropism of MSC‐EVs, CG‐treated mice were intraperitoneally injected with Cy5‐labeled EVs or an equivalent volume of PBS, followed by imaging of the major organs. The results revealed no detectable fluorescent signals in the heart, lungs, liver, spleen, kidneys, or peritoneum of PBS‐treated mice 24 h post‐injection. In contrast, significant fluorescent accumulation was observed in the peritoneum of mice administered with Cy5‐labeled EVs (Figure 2B). To further assess whether MSC‐EVs reached the injured peritoneum, we injected PKH26‐labeled EVs into CG‐treated mice and detected their distribution on the peritoneum via fluorescence microscopy 24 h later. The red PKH26 signal was clearly localized within peritoneal cells, confirming effective EV retention and uptake at the injury site (Figure 2C).

We further evaluated the biocompatibility of MSC‐EVs in mice. Following intraperitoneal administration of MSC‐EVs, the body weights of these mice were recorded every other day. The results demonstrated that body weight fluctuations across all experimental groups remained stable (Figure S1A, Supporting Information). Histological analyses revealed no detectable evidence of toxicity in the heart, liver, spleen, and kidney tissues (Figure S1B, Supporting Information).

We then evaluated the histological changes of murine peritoneum induced by CG. Hematoxylin and eosin (H&E) staining revealed that CG treatment resulted in prominent inflammatory cell infiltration of the peritoneum. Masson's trichrome staining revealed marked thickening of both parietal and visceral peritoneum. In contrast, MSC‐EV‐treated mice exhibited substantially reduced peritoneal thickening and inflammatory cell infiltration (Figure 2D,E; Figure S2A,B, Supporting Information). These findings were further supported by morphometric quantification of peritoneal thickness (Figure 2F). Immunohistochemical staining demonstrated that CG‐induced peritoneal injury was associated with increased expression of α‐smooth muscle actin (α‐SMA), indicating fibroblast activation, and elevated CD31, reflecting neovascularization (Figure 2G,H). Both α‐SMA and CD31 levels were markedly decreased following MSC‐EV treatment. To evaluate functional recovery of peritoneal transport, a modified peritoneal equilibration test was performed. The results showed that CG exposure led to accelerated glucose absorption (decreased D/D0 glucose ratio) and impaired solute clearance (increased D/P creatinine), consistent with compromised membrane function. These abnormalities were partly corrected in MSC‐EV‐treated mice, indicating restoration of peritoneal transport function (Figure 2I,J).

Next, we also analyzed a mouse model of peritoneal inflammation and fibrosis induced by PD fluid (Figure S3A, Supporting Information). H&E staining revealed increased inflammatory infiltration in the peritoneum of PD fluid‐treated mice, which was attenuated by MSC‐EVs treatment (Figure S3B, Supporting Information). Masson's trichrome staining demonstrated reduced deposition of submesothelial collagen in the MSC‐EVs group compared to the PD group (Figure S3C,D, Supporting Information). Additionally, immunohistochemistry confirmed lower protein expression levels of CCL2 and Ly6c in the peritoneum of MSC‐EV‐treated mice relative to the PD model mice (Figure S3E,F, Supporting Information). Concurrently, MSC‐EVs administration also ameliorated peritoneal functional impairment in PD fluid‐treated mice (Figure S3G,H, Supporting Information).

Together, these data demonstrated that MSC‐EVs alleviate peritoneal inflammation, fibrosis, and functional impairment in vivo.

### 2.3 Effect of MSC‐EVs on Immune Cell Composition and Macrophage‐Mesothelial Cell Crosstalk After CG‐Induced Peritoneal Injury

**Figure 3:**
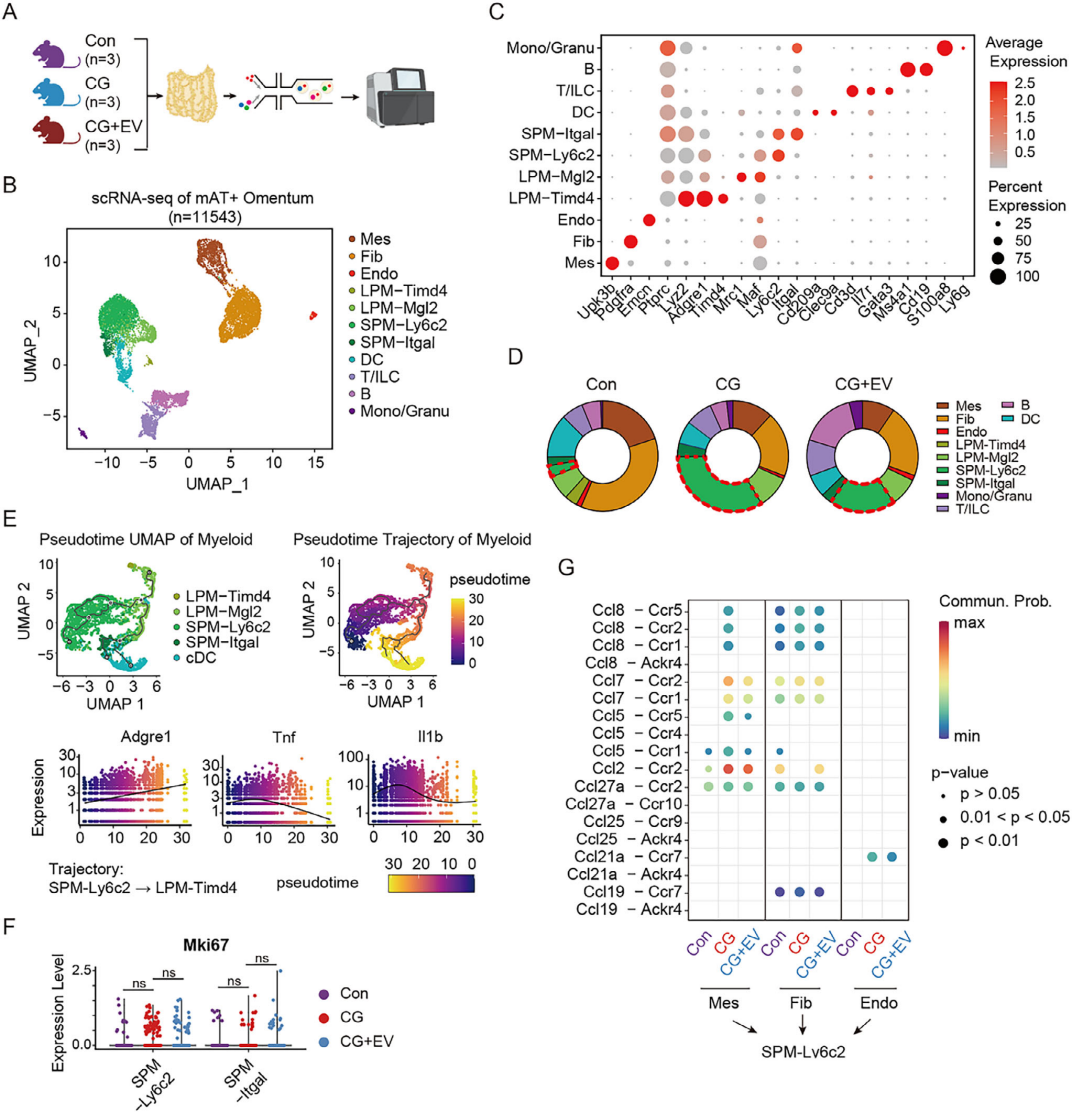
MSC‐EVs modulate immune cell composition and mesothelial‐macrophage crosstalk in peritoneal injury. A) Schematic overview of the scRNA‐seq workflow using peritoneal tissues from the three groups: control (Con), CG‐treated, and CG + MSC‐EV‐treated (CG + EV) mice (n = 3 per group). B) UMAP plot showing clustering of 11 transcriptionally distinct cell populations across all samples. C) A dot plot showing the expression of canonical marker genes used to identify each cell type. Dot size reflects the percentage of expressing cells; color reflects average expression. D) Relative abundance of significant cell populations across experimental groups. Notable changes were observed in mesothelial cells and *Ly6c2*⁺ small peritoneal macrophages (SPMs). The dashed red line denotes the SPM cell subtype. E) Pseudotime analysis of myeloid cell subsets (LPM, SPM, DC) illustrating inflammatory transcriptional states based on *Tnf* and *Il1b* expression. F) Mki67 expression in *Ly6c2*⁺ and *Itgal*⁺ SPMs indicates comparable proliferative capacity across groups. G) A heatmap representing ligand‐receptor interactions between *Ly6c2*⁺ SPMs and structural peritoneal cells. ns: no significance by one‐way ANOVA.

To investigate cellular mechanisms underlying the protective effects of MSC‐EVs, single‐cell RNA sequencing (scRNA‐seq) was performed on peritoneal tissues (mesenteric adipose tissue and omentum, excluding lymph nodes) from three mice per group (Control (Con), CG, and CG + MSC‐EVs (CG + EV)) (**Figure** **3**A). After rigorous quality control, 11 543 cells obtained were grouped into 11 transcriptionally distinct clusters through unsupervised analysis (Figure 3B). These clusters were annotated as mesothelial cells (Mes), fibroblasts (Fib), endothelial cells (Endo), large peritoneal macrophages (*Timd4*⁺ LPM, *Mgl2*⁺ LPM), small peritoneal macrophages (*Ly6c2*⁺ SPM, *Itgal*⁺ SPM), monocyte‐derived dendritic cells (DC), T cells, B cells, and a mixed monocyte/granulocyte population (Mono/Granu), based on marker expression patterns, as shown in Figure 3C.

Comparative analysis revealed striking changes in cell composition following CG‐induced injury. The abundance of mesothelial cells declined markedly in CG‐treated mice. At the same time, the pro‐inflammatory *Ly6c2*⁺ SPM population, previously identified as a key inflammatory subset in the peritoneal cavity,^[^ ^25^ ^]^ was significantly expanded. Notably, MSC‐EV administration partially reversed the shift of *Ly6c2*⁺ SPM population (Figure 3D). The transcriptional changes in immune cells induced by CG stimulation were primarily concentrated in *Ly6c2*⁺ SPM, DC, and *Mgl2*⁺ LPM, with the most significant change observed in *Ly6c2*⁺ SPM (Figure S4A, Supporting Information). Although the changed pathways differed after CG stimulation, all three immune cell types exhibited activation of the innate immune and inflammatory response pathways (Figure S4B–G, Supporting Information). Moreover, pseudotime analysis of myeloid populations (LPM, SPM, DC) indicated a dominant presence of non‐resident (*Adgre1* ^low^) and inflammatory (*Tnf* ^high^ *Il1b* ^high^) signatures across all groups, consistent with the course of acute injury (Figure 3E).

To figure out whether MSC‐EVs had direct regulatory effects on peritoneal immune cells, we performed differential analysis of immune cells between the CG and the CG + EV groups. The results showed no significant reversal of the transcriptome changes caused by CG, while only *Ly6c2*⁺ SPM exhibited a certain number of differentially expressed genes (DEGs) after MSC‐EV treatment (Figure S4H–J, Supporting Information). Pathway enrichment also suggested that MSC‐EV treatment mainly enhanced the antigen presentation ability of *Ly6c2*⁺ SPM, without alleviating its inflammatory effects (Figure S4K, Supporting Information). In addition, the proliferative capacity of *Ly6c2*⁺ and *Itgal*⁺ SPMs, as assessed by Mki67 expression, did not differ significantly among the three groups (Figure 3F), suggesting that altered recruitment, rather than proliferation, accounted for local SPM accumulation.

Biological process enrichment analysis of DEGs of myeloid cells demonstrated that MSC‐EVs significantly reduced the chemotaxis of myeloid cells (Figure S5A, Supporting Information). To explore this further, ligand‐receptor interaction analysis was performed between Ly6c2⁺ SPMs and parenchymal cells. The expression levels of the key chemokines, such as *Ccl2* and *Ccl7*, were significantly mitigated by MSC‐EV treatment in mesothelial cells. In contrast, the receptor expression in *Ly6c2⁺* SPMs didn't change after MSC‐EV treatment (Figure 3G; Figure S5B,C, Supporting Information).

Collectively, these data highlight a key mechanism by which MSC‐EVs reduce inflammatory macrophage recruitment by disrupting Ccl2/Ccl7‐Ccr2‐mediated crosstalk between mesothelial cells and *Ly6c2*⁺ SPMs during peritoneal injury.

### 2.4 MSC‐EVs Reverse Glycolytic and Pro‐Inflammatory Reprogramming in Mesothelial Cells

**Figure 4:**
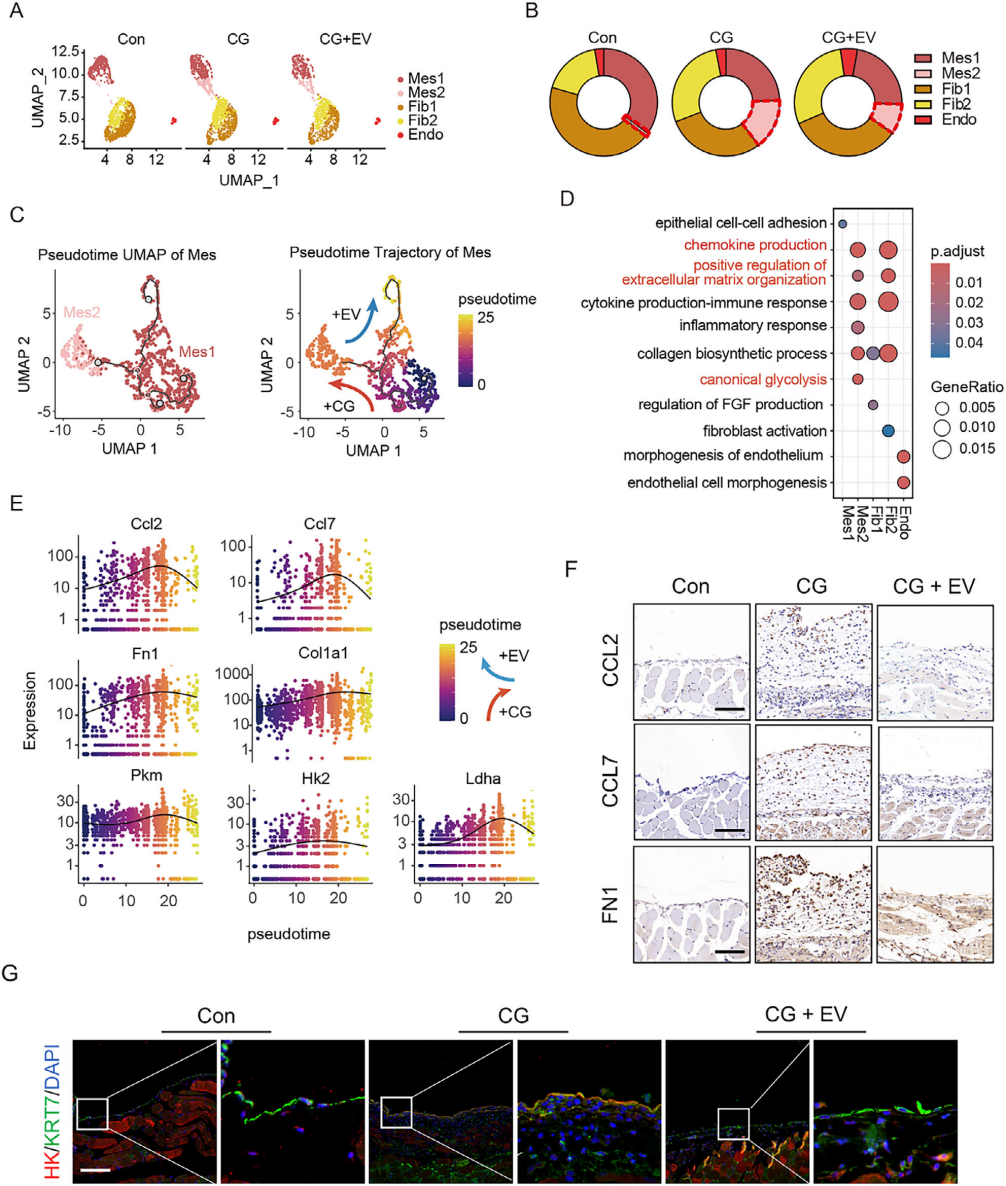
MSC‐EVs reverse glycolytic and pro‐inflammatory reprogramming in mesothelial cells. A) Sub‐clustering of peritoneal parenchymal cells reveals distinct mesothelial and fibroblast subtypes across the experimental groups. B) Relative abundance of each peritoneal parenchymal cell subtype (Mes1, Mes2, Fib1, Fib2, Endo) in Con, CG, and CG + EV groups. The dashed red line denotes the Mes2 cell subtype. C) Pseudotime trajectory analysis showing CG‐induced transition from Mes1 to Mes2, which is partially reversed by MSC‐EV treatment. D) Dot plot showing enriched pathways in cells from the CG and CG + EV groups. The size of the dots reflects gene expression ratios, and their color indicates adjusted *p*‐value. E) Pseudotime trajectory analysis of *Ccl2*, *Ccl7*, *Fn1*, *Col1a1*, *Pkm*, *Hk2*, and *Ldha* expression across the experimental groups. F) Representative immunostaining images for CCL2, CCL7, and FN1 in peritoneal tissue (n = 5). Scale bars, 50 µm. G) Representative immunofluorescence images showing co‐staining of HK and Cytokeratin 7 (KRT7) in mesothelial cells (n = 5): scale bars, 100 µm.

To further characterize the response of parenchymal cells to MSC‐EV treatment, scRNA‐seq analysis was performed, focusing on peritoneal mesothelial cells and fibroblasts. Sub‐clustering revealed two transcriptional states within each population (**Figure** **4**A; Figure S6A, Supporting Information). In particular, the normal mesothelial cell subtype (Mes1) was depleted in CG‐treated mice, whereas an injury‐associated mesothelial cell subtype (Mes2) emerged and predominated. This shift was markedly reduced in MSC‐EV‐treated mice (Figure 4B). Trajectory analysis suggested a CG‐driven transition from Mes1 to Mes2, which was partially reversed following MSC‐EV treatment (Figure 4C). Pathway enrichment of EV‐responsive genes in mesothelial cells revealed significant suppression of glycolysis, chemokine signaling, and extracellular matrix remodeling (Figure 4D; Figure S6B, Supporting Information). Consistently, MSC‐EV treatment downregulated the expression of genes for pro‐inflammatory chemokines (*Ccl2, Ccl7*), extracellular matrix components (*Fn1, Col1a1*), glycolytic enzymes (*Pkm, Hk2*), and lactate dehydrogenase (*Ldha*), and led to a corresponding reduction in CCL2, CCL7, FN1, and HK protein levels (Figure 4E–G).

To validate these findings, bulk RNA sequencing was performed on peritoneal tissue samples collected from mice in the Con, CG, and CG + EV groups. Transcriptomic modularity analysis identified a CG‐upregulated/MSC‐EV‐downregulated gene cluster that was enriched in glycolysis‐related genes (*Hk, Pfk, Ldha*) (Figure S7A–D,F, Supporting Information). Furthermore, we reanalyzed the metabolomic profiles of mesothelial cells with or without PD fluid treatment, revealing elevated levels of various glycolysis‐related metabolites, including lactate, in the injured mesothelial cells stimulated by PD fluid (Figure S7E,F, Supporting Information). Functional validation in vitro also confirmed that MSC‐EVs suppressed glycolytic flux and lactate accumulation in injured mesothelial cells (Figure S8A–F, Supporting Information).

These observations highlighted the capacity of MSC‐EVs to reverse injury‐induced metabolic and inflammatory reprogramming in mesothelial cells, thereby mitigating fibrosis‐promoting conditions in the peritoneal microenvironment.

### 2.5 Inhibition of Lactate Production Attenuates Peritoneal Inflammation and Fibrosis

**Figure 5:**
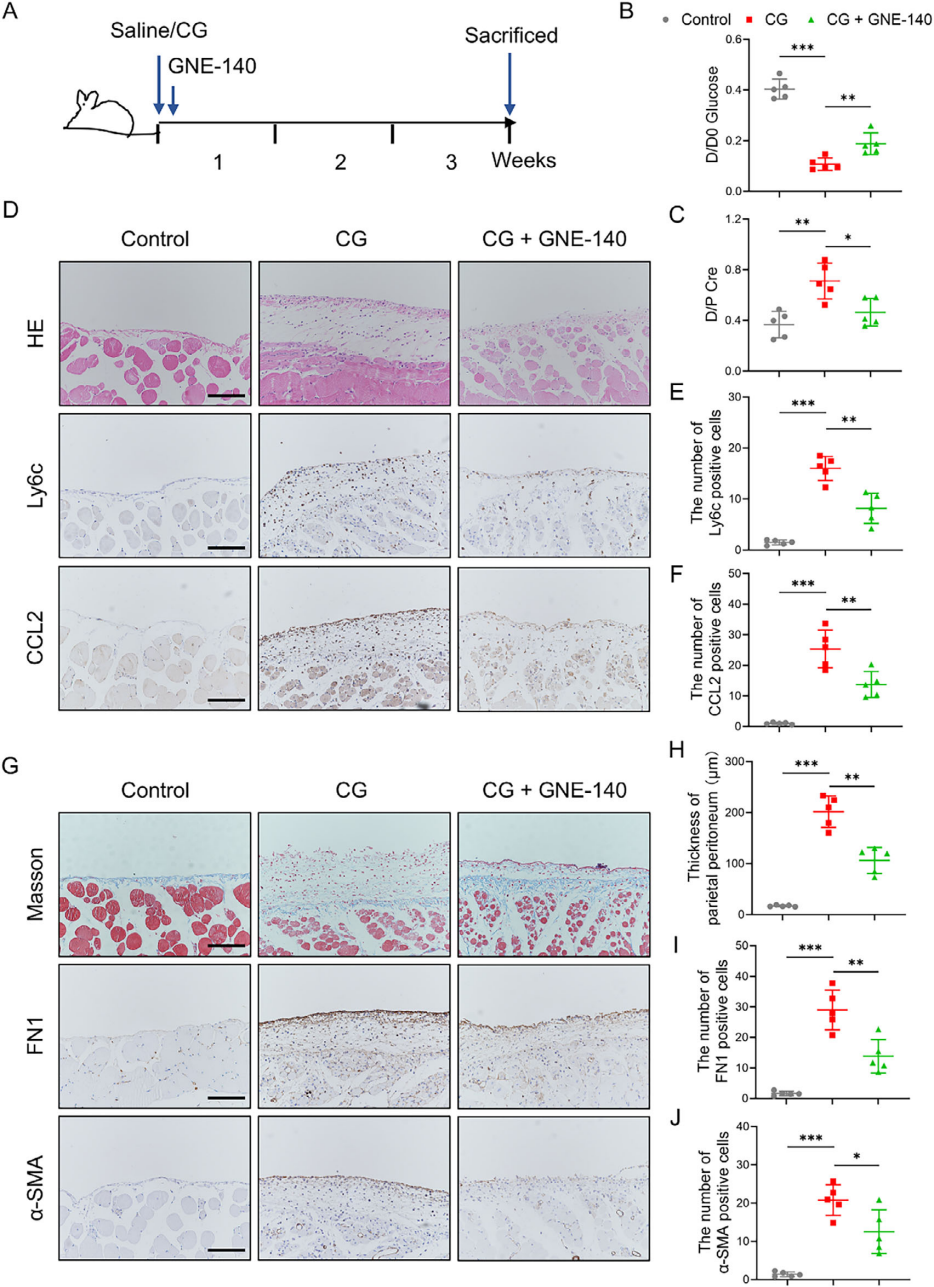
Blocking lactate generation reduces inflammatory and fibrotic responses in CG‐induced peritoneal injury. A) Schematic diagram of the experimental design. Mice received intraperitoneal injections of 0.1% CG or saline (0.01 mL g^−1^ body weight, three times weekly), with or without GNE‐140 treatment (5 µg g^−1^ body weight, three times weekly), starting on day 7. B,C) Modified peritoneal equilibration test showing membrane transport parameters: (B) D/D0 glucose ratio and (C) D/P creatinine ratio (n = 5 per group). D) Representative images of H&E, CCL2, and Ly6c immunostaining in peritoneal tissues (n = 5). Scale bars, 100 µm. E,F) Quantification of Ly6c⁺ (E) and CCL2⁺ (F) cells in peritoneal sections (n = 5). G) Representative Masson's trichrome staining and immunostaining for FN1 and α‐SMA in peritoneal tissues (n = 5). Scale bars, 100 µm. H) Quantification of peritoneal thickness (n = 5). I,J) Quantification of FN1⁺ (I) and α‐SMA⁺ (J) cells in the peritoneum (n = 5). Data are presented as mean ± SD. ^*^ *p* <0.05, ^**^ *p* <0.01, ^***^ *p* <0.001 by one‐way ANOVA.

Our prior work established that hyperglycolysis in mesothelial cells plays a crucial role in promoting peritoneal fibrosis, and that inhibiting glycolytic enzymes alleviates the progression of fibrosis.^[^ ^17^ ^]^ Here, we confirmed that chronic CG exposure resulted in a markedly increased glycolytic activity and lactate accumulation in peritoneal tissues and that these effects were substantially mitigated by MSC‐EV administration (Figure S9, Supporting Information). To further examine the functional role of lactate in CG‐induced peritoneal injury, the production of lactate was abolished using GNE‐140, a selective LDHA/B inhibitor. To this end, mice subjected to CG exposure were administered GNE‐140 (5 µg g^−1^ body weight) intraperitoneally three times a week for 14 days, starting on day 2 after CG initiation (**Figure** **5**A). It turned out that GNE‐140 significantly improved peritoneal transport parameters, increasing the D/D0 glucose ratio and reducing the D/P creatinine ratio, thus indicating restored peritoneal membrane function (Figure 5B,C). Moreover, GNE‐140 administration diminished inflammatory cell infiltration and reduced peritoneal thickening (Figure 5D,G,H). In addition, GNE‐140 administration led to a marked decrease in *Ly6c*⁺ macrophage infiltration (Figure 5E), CCL2 expression (Figure 5F), as well as in FN1 and α‐SMA expression (Figure 5I,J).

Subsequently, we employed a mouse model treated with PD fluid to investigate whether lactate regulates the progression of peritoneal inflammation and fibrosis. The mice received daily intraperitoneal injection of PD fluid for six consecutive weeks. Beginning on day 2 post‐PD fluid initiation, mice were treated with either GNE‐140 (5 µg g^−1^ body weight) or saline (Figure S10A, Supporting Information). As anticipated, chronic PD fluid exposure promoted inflammatory cell infiltration and submesothelial collagen deposition in the peritoneum. Notably, GNE‐140 treatment effectively attenuated peritoneal inflammation and collagen formation, as well as suppressed PD fluid‐induced upregulation of CCL2 and Ly6c expression in peritoneal tissues (Figure S10B–F, Supporting Information). Furthermore, the impaired peritoneal function was also ameliorated by GNE‐140 (Figure S10G,H, Supporting Information).

Collectively, these findings underscore the pathogenic role of lactate in promoting peritoneal inflammation and fibrosis and demonstrate that pharmacological inhibition of lactate production effectively preserves peritoneal structure and function.

### 2.6 MSC‐EVs Attenuate CG‐Induced Peritoneal Inflammation Through Epigenetic Modulation of H3K18 Lactylation‐CCL2 Axis

**Figure 6:**
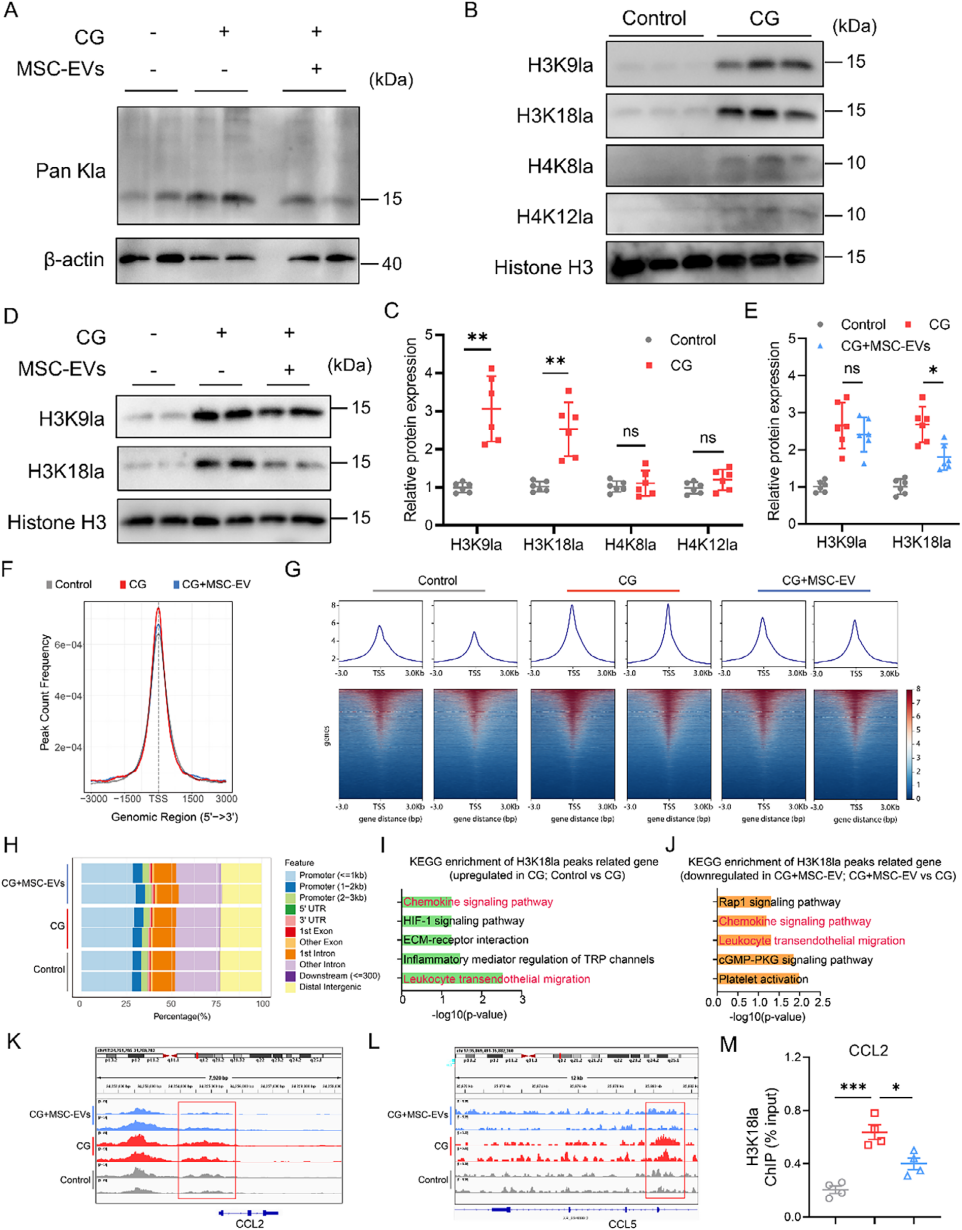
MSC‐EVs attenuate CG‐induced peritoneal inflammation through epigenetic modulation of H3K18la‐CCL2 axis. A) Representative immunoblotting showing Pan Kla protein expression in the peritoneum of Control, CG, and CG + MSC‐EVs groups. B,C) Representative immunoblotting (B) and quantitative data (C) showing the H3K9la, H3K18la, H4K8la, and H4K12la expressions in the peritoneum of Control and CG groups (n = 6). D,E) The protein levels of H3K9la and H3K18la in the peritoneum of Control, CG, and CG + MSC‐EVs groups were determined by western blot (n = 6). F) The read count frequency in the selected range around TSS in Control, CG, and CG + MSC‐EV groups. G) Heatmaps of H3K18la CUT&Tag signals were visualized by deepTools in Control, CG, and CG + MSC‐EV groups. These findings were ordered by signal strength. H) Genome‐wide distribution of the differential H3K18la‐binding peaks. I) KEGG enrichment analysis of the upregulated peaks (upregulated in the CG group, compared to the Control) upon the binding of H3K18la to the candidate genes. J) KEGG enrichment analysis of the downregulated peaks (downregulated in the CG + MSC‐EV group, compared to the CG) upon the binding of H3K18la to the candidate genes. K,L) Genome browser track analysis reveals the H3K18la lactylation levels in the CCL2 (K) and CCL5 (L) binding regions across the three groups. M) The promoters of the CCL2 genes that bind to H3K18la after the 0 nm and 20 mm sodium lactate, and MSC‐EVs treatment for 24 h were detected using ChIP‐qPCR assays (n = 4). Data are presented as mean ± SD. ^*^ *p* <0.05, ^**^ *p* <0.01, and ^***^ *p* <0.001 by one‐way ANOVA or Student's *t*‐test. ns: no significance.

Given that histone lactylation represents a recently characterized epigenetic mechanism linking metabolism to gene regulation,^[^ ^20^ ^]^ we sought to determine whether this modification could contribute to CG‐induced peritoneal inflammation and fibrosis. Western blot analysis revealed a marked increase in global lysine lactylation (Pan‐Kla) in peritoneal tissues from mice exposed to CG, and this effect appeared to be less after the MSC‐EV treatment (**Figure** **6**A). To identify specific histone lysine residues affected by CG exposure, we screened several lactylation sites. We found that H3K9la, H3K18la, H4K8la, and H4K12la were all elevated in response to CG, with the most potent induction observed for H3K18la (Figure 6B,C). Interestingly, MSC‐EVs selectively reduced H3K18la levels without significantly affecting H3K9la (Figure 6D,E), suggesting site‐specific epigenetic regulation. To investigate whether lactate directly drives H3K18la and chemokine expression in mesothelial cells, we treated cells in vitro with 20 mm sodium lactate. Lactate treatment markedly increased both H3K18la and CCL2 protein levels. Whereas co‐treatment with MSC‐EVs abrogated these effects (Figure S11A–D, Supporting Information). At the transcriptomic level, treatment with MSC‐EVs suppressed the CCL2 expression in mesothelial cells induced by CG, while its impact on CCL7 expression was not significant (Figure S11E,F, Supporting Information).

To elucidate the regulatory role of histone lactylation on gene expression in mesothelial cells, we performed CUT&Tag sequencing using an anti‐H3K18la antibody on mesothelial cells treated with CG or MSC‐EVs. The results revealed that, compared with the control group, the CG group exhibited an enrichment of H3K18la peaks, with ≈30% of the H3K18la binding peaks located within promoter regions (≤3 kb) (Figure 6G,H). Following MSC‐EV treatment, the enrichment of H3K18la peaks was reduced compared to the CG group (Figure 6F,G). To investigate the epigenetic regulatory function of H3K18la in injured mesothelial cells, KEGG pathway analysis was performed on the target genes associated with H3K18la peak binding sites. The results indicated that genes associated explicitly with H3K18la in the CG group were significantly enriched in pathways related to the chemokine signaling pathway and leukocyte transendothelial migration, compared with the control. Interestingly, MSC‐EVs treatment suppressed these enrichments (Figure 6I,J). Based on our previous experimental findings, we focused on chemokines. Specifically, the H3K18la signal was significantly enriched at the promoters of CCL2 and CCL5 in the CG group, compared with the control. This significant enrichment was attenuated in the MSC‐EV‐treated group (Figure 6K,L). To confirm whether H3K18la directly regulates CCL2 transcription, we performed chromatin immunoprecipitation (ChIP) assays. H3K18la enrichment at the CCL2 promoter was significantly increased after lactate treatment and diminished following MSC‐EV administration, indicating a functional link between metabolic reprogramming, histone modification, and inflammatory gene expression (Figure 6M).

To investigate the pivotal role of CCL2 in the regulation of macrophage chemotaxis by MSC‐EV‐treated mesothelial cells, we first quantified the expression of CCL2 in the conditioned medium of these cells. As anticipated, CG stimulation significantly enhanced CCL2 secretion by mesothelial cells, an effect that was markedly suppressed by MSC‐EV treatment (Figure S12A, Supporting Information). Using a non‐contact co‐culture system of mesothelial cells and macrophages, we found that CG‐injured mesothelial cells promoted the migration of macrophages. Notably, neutralization of CCL2 partially recapitulated the inhibitory effect of MSC‐EVs on the chemotactic capacity of injured mesothelial cells toward macrophages (Figure S12B–D, Supporting Information).

Finally, we evaluated the expression of H3K18la in peritoneal tissues from a long‐term PD patient. Immunohistochemical staining revealed a significant upregulation of H3K18la expression in the peritoneal tissues from patients undergoing long‐term PD compared with the control group, which exhibited a consistent trend with the elevated expression of CCL2. The results suggest that H3K18la may contribute to the regulation of PD‐induced peritoneal inflammatory processes (Figure S13, Supporting Information).

Taken together, our findings revealed that MSC‐EVs alleviate CG‐induced peritoneal inflammation by disrupting the lactate/H3K18la/CCL2 axis and established H3K18la as a key epigenetic mediator of peritoneal fibrogenesis.

## 3 Discussion

Although PD is a valuable and viable form of renal replacement therapy, its wider dissemination worldwide is hampered by concerns about the durability of the peritoneum as a dialysis membrane. This is related to the fact that in some patients, the peritoneum may undergo adverse fibrotic thickening, leading to peritoneal dysfunction and ultrafiltration failure.^[^ ^26^ ^]^ The lack of effective pharmacological intervention to date has prompted substantial research efforts aimed at preventing or reversing peritoneal fibrosis. Here, we present data suggesting that extracellular vesicles derived from human bone marrow mesenchymal stem cells could be an effective option for addressing this problem.

We found that intraperitoneal administration of human MSC‐EVs markedly attenuated inflammation and fibrogenesis in the mouse model of peritoneal injury. This highlights the translational potential of MSC‐EVs and their favorable immunological profile, confirming earlier findings that xenogeneic application of human EVs across multiple disease models retains their therapeutic efficacy without eliciting immune rejection.^[^ ^27^, ^28^ ^]^

Fibrotic thickening of the peritoneum is likely to result from sustained and/or unresolved peritoneal inflammation.^[^ ^29^ ^]^ We found that MSC‐EVs could disrupt the vicious cycle of injury‐induced inflammation and recovery by downregulating pro‐inflammatory mediators and promoting the restoration of mesothelial homeostasis. Through scRNA‐seq, we revealed that mesothelial cell‐macrophage crosstalk, involving chemokine signaling, plays a central role in the fibrotic remodeling of the peritoneum. We observed that injured mesothelial cells promoted the recruitment of *Ly6c2*⁺ macrophages through upregulation of specific chemotactic ligands CCL2 and CCL7.

Moreover, consistent with previous studies,^[^ ^30^ ^]^ we observed that after CG exposure, mesothelial cells underwent pronounced metabolic reprogramming, characterized by enhanced glycolytic flux and lactate accumulation. Being not only a metabolic by‐product, lactate can also act as a signaling molecule that influences inflammatory responses. In line with prior observations,^[^ ^31^ ^]^ our data showed that lactate induced CCL2 expression in mesothelial cells, further exacerbating inflammatory infiltration. Moreover, we provide mechanistic insight into lactate's downstream effects through histone lysine lactylation, a recently described epigenetic modification that links metabolism to gene regulation.^[^ ^32^ ^]^

Among several histone lactylation sites, H3K18la emerged as a remarkably responsive and functionally relevant mark during the development of peritoneal fibrosis. Our results demonstrate that H3K18la is significantly elevated in CG‐treated tissues and directly enriched at the CCL2 promoter, facilitating transcriptional activation of this key chemokine. Notably, MSC‐EV treatment not only reduced lactate levels but also selectively suppressed H3K18la, thereby disrupting this metabolic‐epigenetic‐inflammatory axis. These findings extend previous knowledge from hepatic fibrosis and immune regulation^[^ ^24^, ^33^ ^]^ by establishing H3K18la as a mediator of inflammation in mesothelial cells.

Interestingly, we found that GNE‐140, a pharmacological inhibitor of LDHA/B, could recapitulate many anti‐inflammatory and anti‐fibrotic effects of MSC‐EVs. However, the therapeutic rationale for using MSC‐EVs over small‐molecule inhibitors lies in their multimodal specificity. Unlike GNE‐140, which globally inhibits glycolysis and may carry systemic side effects, MSC‐EVs selectively modulate the local peritoneal microenvironment through targeted delivery of bioactive cargo. These include miRNAs, proteins, and lipids that collectively regulate gene expression, immune signaling, and metabolic activity without perturbing systemic homeostasis. Thus, MSC‐EVs offer a precision‐targeted therapeutic strategy with broader regulatory potential and reduced risk of off‐target effects.

Nevertheless, our study has several limitations. First, our study primarily focused on mesothelial cells and macrophages, which constitute the largest cell populations in the peritoneum. However, MSC‐EVs may also act on other cell types, including peritoneal fibroblasts, vascular endothelial cells, and lymphocytes. Future studies employing comprehensive single‐cell multi‐omics and lineage tracing will be essential to delineate cell‐type‐specific mechanisms of MSC‐EV activity. Additionally, the current mapping of histone lactylation is likely incomplete. Other unexplored lysine sites may also contribute to peritoneal pathogenesis and remain to be functionally validated. Finally, while MSC‐EVs deliver a rich cargo of regulatory molecules, the precise molecular effectors responsible for modulating the lactate‐H3K18la‐CCL2 axis remain unidentified. Multi‐omics integration and high‐throughput screening approaches are needed to pinpoint critical EV components driving therapeutic effects.

In conclusion, our findings identify lactate‐driven H3K18 histone lactylation as a key epigenetic mechanism promoting peritoneal inflammation and fibrogenesis. We demonstrate that MSC‐EVs alleviate these pathological processes by disrupting the H3K18la‐CCL2 axis and crosstalk between mesothelial cells and *Ly6c2*⁺ macrophages. This observation establishes a novel metabolic‐epigenetic link and a robust mechanistic foundation for the development of MSC‐EV‐based therapies in the clinical management of peritoneal fibrosis during PD.

## 4 Experimental Section

### Cell Culture

Human bone marrow‐derived MSCs were obtained from Lonza (Lonza PT‐2501). MSCs were cultured in MEM Alpha medium supplemented with 15% fetal bovine serum (FBS), 1% penicillin/streptomycin (PS), and 1% glutamine. The THP‐1 cell line was purchased from ATCC. THP‐1 was cultured in RPMI‐1640 containing 10% FBS and 1% PS.

### Flow Cytometry

MSC purity was validated by flow cytometric analysis of surface markers (CD105, CD90, CD34, and CD45), with all antibody sources and catalog numbers provided in Table S1 (Supporting Information). Briefly, MSCs were resuspended in Cell Staining Buffer and centrifuged at 350 × g for 5 min. After discarding the supernatant, MSCs were pre‐incubated with Fc Receptor Blocking Solution for 5 min at room temperature. Next, MSCs were incubated with the conjugated fluorescent compound on ice in the dark for 20 min. Following washing with Cell Staining Buffer, cell pellets were resuspended and prepared for flow cytometric analysis.

### EV Isolation and Identification

To generate EV‐enriched conditioned medium, MSCs at passages 4–6 were cultured to 70–80% confluency. Before supernatant collection, the culture medium was replaced with EV‐depleted FBS‐supplemented medium. MSCs were cultured for 48 h before harvesting supernatants.

EVs were isolated from the cell supernatants via ultracentrifugation as previously described.^[^ ^34^ ^]^ Briefly, MSC supernatants were subjected to differential centrifugation: 5 min at 300 × g, 20 min at 2000 × g, and 30 min at 10 000 × g. Clarified supernatants were filtered through 0.22 µm filters (Merck Millipore, SLGP033RB) and subsequently subjected to ultracentrifugation (100 000 × g, 90 min) using an SW 28 Ti rotor (Beckman Coulter, USA). Pelleted EVs were resuspended in cold PBS, re‐pelleted under identical ultracentrifugation conditions, and finally resuspended in 100 µL PBS. Isolated EVs were then characterized by NTA, TEM, and immunoblotting of canonical EV markers (as detailed in subsequent sections).

### Transmission Electron Microscopy (TEM)

EV preparations were deposited onto copper grids and incubated for 1 min at ambient temperature. Excess liquid was removed before chemical fixation with 2.5% glutaraldehyde for 10 min. Staining was performed by applying 2% uranyl acetate solution onto the grids. Specimens were visualized under a TEM (Hitachi, Japan) operated at 100 kV, with micrographs systematically captured at various magnifications across multiple fields.

### Nanoparticle Tracking Analysis (NTA)

MSC‐EV quantification and dimensional characteristics were analyzed using the NanoSight NS300 analytical system (Malvern, UK). Following appropriate dilution with PBS to yield final concentrations between 10^7^ and 10^8^ particles mL^−1^ in 0.8 mL suspension, specimens were slowly introduced into the measurement chamber. During analytical procedures, particle diffusion characteristics and Brownian movement were monitored through integrated video capture technology. Experimental conditions were maintained at 24 °C ± 1 °C, coupled with optical detection settings at camera sensitivity levels 13–15. The measurements from triplicate recordings were computationally processed, ultimately generating nanoparticle population statistics including EV diameter (nm) and concentration (particles mL^−1^) using a detection threshold of five.

### Western Blot

Cellular proteins and peritoneal tissue extracts were obtained through lysis with RIPA buffer (Biosharp, BL‐504‐A) supplemented with dual protease‐phosphatase inhibitors, maintaining constant ice‐cooling. Following complete homogenization, the lysates were centrifuged at 12 000 × g and 4 °C for 10 min to isolate the soluble fractions. Protein quantification was subsequently performed using a bicinchoninic acid assay system. Lysates were mixed with loading buffer and heat‐denatured at 95 °C for 5 min before electrophoretic analysis. For immunoblotting procedures, 15 µg of protein underwent electrophoretic separation through 10–12.5% SDS‐PAGE gels before being transferred onto PVDF membranes. Membranes were treated with 5% milk or 5% BSA blocking buffer for 1 h at room temperature, then exposed to primary antibodies overnight at 4 °C. Following extensive TBST washing, membranes were incubated with HRP‐linked species‐specific immunoglobulins (Boster, BA1054 or BA1050), and then underwent enhanced chemiluminescent detection. The primary antibodies used in this study are listed in Table S1 (Supporting Information).

EV samples were subjected to immediate downstream processing (protein denaturation and electrophoretic separation) following protein quantification, omitting the conventional protein extraction protocol.

### EV Labeling

EVs were labeled by incubation with 10 µm of DiO fluorescent dyes (Invitrogen, V‐22 886) or PKH26 fluorescent dyes (Sigma‐Aldrich, MINI26) at 37 °C for 30 min. Post‐labeling, EV suspensions were subjected to three PBS washing cycles followed by ultracentrifugation (100 000 × g, 90 min, 4 °C) to eliminate unbound dyes. Purified EVs were reconstituted in 100 µL PBS. Biodistribution analysis was performed in mice through intraperitoneal injection (100 µg EVs per mouse) and mesothelial cell uptake assays (30 µg mL^−1^ EVs).

### EV Biodistribution

CG‐induced model mice were intraperitoneally injected with Cy5‐labeled EVs (100 µg EVs per mouse) or an equivalent volume of PBS. After 24 h, whole‐body scanning was performed using an IVIS® Lumina III imaging system (PerkinElmer). Subsequently, surgical dissection was conducted to isolate the heart, liver, spleen, kidneys, lungs, parietal peritoneum, mesentery, and greater omentum for ex vivo organ imaging.

### Mouse Model and Sample Collection

All experimental animals were housed in the Specific Pathogen‐Free facility at South China Agricultural University Laboratory Animal Center, with protocols approved by the Institutional Animal Care and Use Committee (2024H035) in strict compliance with ARRIVE guidelines. Peritoneal fibrosis was induced in 8‐week‐old male C57BL/6 mice through intraperitoneal administration of 0.1% CG (Macklin, C832370) (0.01 mL g^−1^ body weight) three times weekly for three consecutive weeks. To evaluate the therapeutic potential of MSC‐EVs, mice were randomized into three experimental cohorts: a saline control, a CG model, and a CG + MSC‐EVs group (5 µg g^−1^ body weight, administered intraperitoneally three times weekly starting from week 2). Terminal procedures included plasma collection, harvesting of visceral/parietal peritoneal tissue (n = 7 per group), and peritoneal equilibration testing. To induce the PD‐associated peritoneal fibrosis, intraperitoneal injection of 4.25% PD fluid (0.1 mL g^−1^ body weight, daily) was given for six consecutive weeks. To assess the therapeutic efficacy of MSC‐EVs on PD‐associated peritoneal fibrosis, mice were randomly allocated into three groups (n = 5 per group): Control, PD, and PD + MSC‐EVs. Starting on day 14 of the model establishment, MSC‐EVs were administered at the exact dosage, frequency, and route of delivery as described previously. At the experimental endpoint, specimens were processed according to the protocol mentioned above. For lactate metabolism studies, age‐matched C57BL/6 mice were allocated into: Saline, CG, CG + GNE‐140 (MCE, HY‐100742A) (LDHA inhibitor, 5 µg g^−1^ body weight, intraperitoneal administration, three times weekly from day 2); Saline, PD fluid, PD fluid + GNE‐140 (5 µg g^−1^ body weight, intraperitoneal administration, three times weekly from day 2). Tissue specimens (n = 5/group) were processed using identical protocols as aforementioned.

### Modified Peritoneal Equilibration Test

Peritoneal membrane permeability was assessed using an optimized peritoneal equilibrium test. 3 mL of a 4.25% glucose‐based peritoneal dialysis solution was administered intraperitoneally. Dialysate and paired blood samples were collected 2 h post‐injection for quantitative analysis. Key transport parameters were calculated as follows: the glucose absorption ratio (D/D0 glucose), comparing 2‐h dialysate concentrations with baseline concentrations, and the creatinine dialysate‐to‐plasma ratio (D/P creatinine), derived from 2‐h dialysate and concurrent plasma measurements.

### Histology and Immunohistochemical Staining

Histopathological evaluation was performed using Masson's trichrome staining and immunohistochemical (IHC) analysis according to established protocols. Peritoneal tissue samples were immersion‐fixed in 4% paraformaldehyde at ambient temperature for 24–48 h. Following paraffin embedding, serial sections (4 µm thickness) were prepared using a rotary microtome for histological evaluations. For IHC characterization, the following primary antibodies were employed: anti‐FN1, anti‐α‐SMA, anti‐CCL2/MCP‐1, anti‐CCL7, anti‐Ly6c, and anti‐H3K18la.

### Immunofluorescence Staining of Frozen Sections

Tissue sections were equilibrated at ambient temperature for 20 min before permeabilization with 0.3% Triton X‐100 for 10 min. After delineating regions using a hydrophobic barrier pen, nonspecific binding was blocked with 5% BSA for 30 min. Following the removal of the blocking buffer, sections were incubated with primary antibodies at 4 °C for 16 h. After incubation, the slides underwent PBST washes before being incubated for 1 h with species‐matched Alexa Fluor‐conjugated secondary antibodies. Nuclear staining was achieved using a DAPI‐containing antifade mounting medium, and fluorescence signals were captured via confocal microscopy.

### CUT&Tag

The CUT&Tag procedure was performed according to the manufacturer's protocol using a hyperactive in situ ChIP Library Prep Kit (pG‐Tn5) designed for Illumina. After treatment, cells were harvested and attached to concanavalin A‐coated beads. Subsequently, samples were treated with a primary antibody targeting H3K18la, then with corresponding secondary antibodies. Processed specimens were combined with pA‐Tn5 transposase. Following activation of Tn5‐driven transposition, genomic DNA was isolated and amplified to generate sequencing libraries. Purification was carried out with VAHTS DNA Clean beads. Library concentration was determined with the VAHTS Library Quantification Kit compatible with Illumina, and paired‐end sequencing was performed on an Illumina NovaSeq system. LC‐Bio Technology conducted all sequencing procedures.

### Bulk RNA Sequencing for Peritoneal Tissues

Peritoneum samples from Control, CG, and CG + MSC‐EVs (CG + EV) groups (n = 5 per group) were collected. Total RNA was isolated, and its quality was evaluated using an Agilent 2100 Bioanalyser. cDNA libraries were prepared and sequenced on an Illumina Genome Analyzer platform.

### Metabolomics Sequencing Analysis

Metabolomic data were obtained from our previous study and preprocessed following the analytical pipeline established therein.^[^ ^17^ ^]^ Principal component analysis (PCA) was performed using the FactoMineR R package, and data visualization was conducted with the ggplot2 package.

### Single‐Cell RNA Sequencing Analysis

Single‐cell transcriptomic profiling data from three normal mouse peritoneum, CG‐treated mouse peritoneum, and CG + EV‐treated mouse peritoneum were processed with the Seurat analytical pipeline (version 3.2.3) within the R statistical framework. Initial quality filtering and normalization procedures were implemented through Seurat's standard workflow. Technical batch variations were harmonized via Harmony integration (R package, version 0.1.0). Cellular subpopulations were identified through nearest‐neighbor graph‐based clustering utilizing FindNeighbors and FindClusters algorithms. Dimensionality reduction was achieved through Uniform Manifold Approximation and Projection (UMAP) visualization, with cluster identities determined through established lineage‐specific biomarkers. Differential expression analysis among peritoneal parenchymal cells from the three groups was performed using Seurat's FindAllMarkers function. Cell communication analysis was conducted using the CellChat R package (version 1.5.0).

### Human Sample Collections

Human peritoneal tissues were sourced from inguinal hernia patients with preserved renal function and individuals undergoing PD at The Third Affiliated Hospital of Sun Yat‐sen University. The study protocol was approved by the hospital's Ethics Committee ([2022]02‐268‐01). Informed consent was acquired from all participants. Primary mesothelial cells were isolated from omental tissue obtained from ten donors.

### Primary Culture and Treatment of Human Peritoneal Mesothelial Cells

Human peritoneal mesothelial cells were isolated from omental tissue specimens (2 × 2 cm) through enzymatic digestion. Freshly excised tissues were rinsed in ice‐cold HBSS, minced into 1 mm^3^ fragments, and subjected to sequential digestion with 0.25% trypsin‐EDTA at 37 °C for 20 min under gentle agitation. The enzymatic reaction was quenched with 10% FBS, followed by filtration through a 70 µm cell strainer. The filtrate was centrifuged at 300 × g for 5 min to pellet cellular components. Mesothelial cells were purified via positive selection using an anti‐UPK3B antibody coupled with fluorescence‐activated cell sorting. Isolated cells were expanded in Earle's M199 medium supplemented with 15% FBS, 1% PS, and 1% insulin‐transferrin‐selenium. For functional investigations, confluent mesothelial cells were exposed to 20 mm sodium lactate, 0.1% CG, 10 ng mL^−1^ recombinant human TGF‐β1, or 30 µg mL^−1^ MSC‐EVs.

### RNA Isolation and Quantitative Real‐Time PCR (Real‐Time qPCR)

Total RNA was isolated from samples using the RNA Quick Purification kit (ESscience, RN001). Subsequent reverse transcription was carried out with the PrimeScript RT reagent kit (Takara, RR047A). qPCR analysis was then performed on a Roche LightCycler 480 system, following the manufacturer's standard protocol. The resulting cycle threshold (Ct) values were normalized to the reference gene β‐actin. Relative gene expression levels were quantified by applying the −ΔΔCt method. Briefly, the ΔCt for each sample was determined by subtracting the mean β‐actin Ct value from the target gene Ct value. Next, the ΔΔCt value was calculated by subtracting the average ΔCt of the control group from the individual sample ΔCt. Finally, relative expression was derived as 2^−ΔΔCt. Primer sequences were detailed in Table S2 (Supporting Information).

### Seahorse Analysis

The Seahorse XFe24 sensor cartridges were pre‐hydrated with 200 µL of calibration solution per well and equilibrated in a non‐CO_2_ incubator at 37 °C for 24 h before assay initiation. On the experimental day, XFe24 cell culture microplates were pre‐coated with Cell‐Tak adhesive at ambient temperature for 20 min. Conditioned cells (2 × 10^5^ cells/well) were resuspended in assay‐specific medium and seeded into the pretreated microplates. Following a 30‐min incubation in the non‐CO_2_ incubator, the supernatant was aspirated, and the wells were rinsed with pre‐warmed XF assay medium. Cells were then maintained in 180 µL of XF assay medium for 60 min. Next, pharmacological modulators (Glucose, Oligomyci, and 2‐DG) were loaded into designated ports. The hydrated sensor cartridge was calibrated and docked to the cell culture plate for real‐time extracellular acidification rate (ECAR) monitoring using the Agilent Seahorse XFe24 Analyzer.

### Chromatin Immunoprecipitation (ChIP)‐PCR

The ChIP assay was conducted according to established protocols using the SimpleChIP Enzymatic Chromatin IP Kit (Cell Signaling Technology, 9003). Briefly, formaldehyde‐fixed cells underwent sequential processing involving cross‐link reversal, enzymatic chromatin digestion, and ultrasonic fragmentation to generate nucleoprotein complexes of 200–500 bp in length. Immunoprecipitation was performed by incubating chromatin lysates with anti‐H3K18la antibody (PTM Bio, PTM‐1427RM) at 4 °C for 16 h with constant rotation. Protein‐DNA complexes were subsequently captured using magnetic bead conjugation during a 4‐h ambient temperature incubation. The precipitated DNA fragments containing the CCL2 promoter were quantified by qPCR using the primers described in Table S3 (Supporting Information).

### Enzyme‐Linked Immunosorbent Assay (ELISA)

The concentration of CCL2 in the mesothelial cell conditioned medium was measured using a human CCL2 ELISA kit, strictly following the manufacturer's instructions.

### Macrophage Migration

Cell migration assays were performed using 24‐well Transwell plates (Corning, USA) with an eight µm pore size. THP‐1 cells (2 × 10^5^ cells/well) were seeded in the upper chamber and differentiated into macrophages following 100 ng mL^−1^ phorbol 12‐myristate 13‐acetate (PMA, Sigma, P1585) treatment for 48 h. Meanwhile, mesothelial cells (1 × 10^5^ cells/well) were cultured in the lower chamber and treated for 24 h under one of the following conditions: CG, CG + MSC‐EVs, CG + anti‐IgG, or CG + anti‐CCL2 antibody (MCE, HY‐P99188). After treatment, the medium in the lower chamber was replaced with fresh complete M199 medium, while the upper chamber received RPMI 1640 medium containing 1% FBS. The cells were then co‐cultured for an additional 24 h. Migrated cells were stained with crystal violet and quantified.

### Statistical Analysis

Statistical analyses were performed using GraphPad Prism 9 and RStudio (v3.6.3). Details of individual tests, including the number of replications performed (*n*) and the reported error as mean ± S.D., are outlined within each Figure legend. Inter‐group comparisons were conducted through an unpaired Student's *t*‐test for parametric data between two cohorts. One‐way ANOVA with Bonferroni post hoc correction was used for multi‐group analyses. A value of *p* <0.05 was considered statistically significant (^*^ *p* <0.05, ^**^ *p* <0.01, and ^***^ *p* <0.001).

## Conflict of Interest

The authors declare that they have no competing interests.

## Author Contributions

Q.H, Y.S., P.K., and J.S. contributed equally to this work. Q.H., Z.H., and H.P. conceived and designed the study. P.K., H.Z., Q.S., and Z.H. performed in vitro experiments. Q.H., J.S., and D.G. performed in vivo experiments. Y.S. analyzed the scRNA‐seq, bulk RNA‐seq, and metabolomics data. Q.H. wrote the manuscript. Z.H., H.P., W.J., and C.L. revised the manuscript.

## Supporting information



Supporting Information

## Data Availability

The data that support the findings of this study are available from the corresponding author upon reasonable request.
