# Supplementary material for: Mesenchymal Stem Cell‐Derived Extracellular Vesicles Modulate the Course of Peritoneal Inflammation Through Metabolic and Epigenetic Regulation
Source: Adv Sci (Weinh). 2025 Nov 26;13(4):e08645. doi: 10.1002/advs.202508645 (PMC12822385; doi:10.1002/advs.202508645)
Supplement: Supplementary file 1 — Supporting Information [file ADVS-13-e08645-s001.docx]

**Supplemental Materials for**

Mesenchymal stem cell-derived extracellular vesicles modulate the course of peritoneal inflammation through metabolic and epigenetic regulation

**Supplemental Figures**


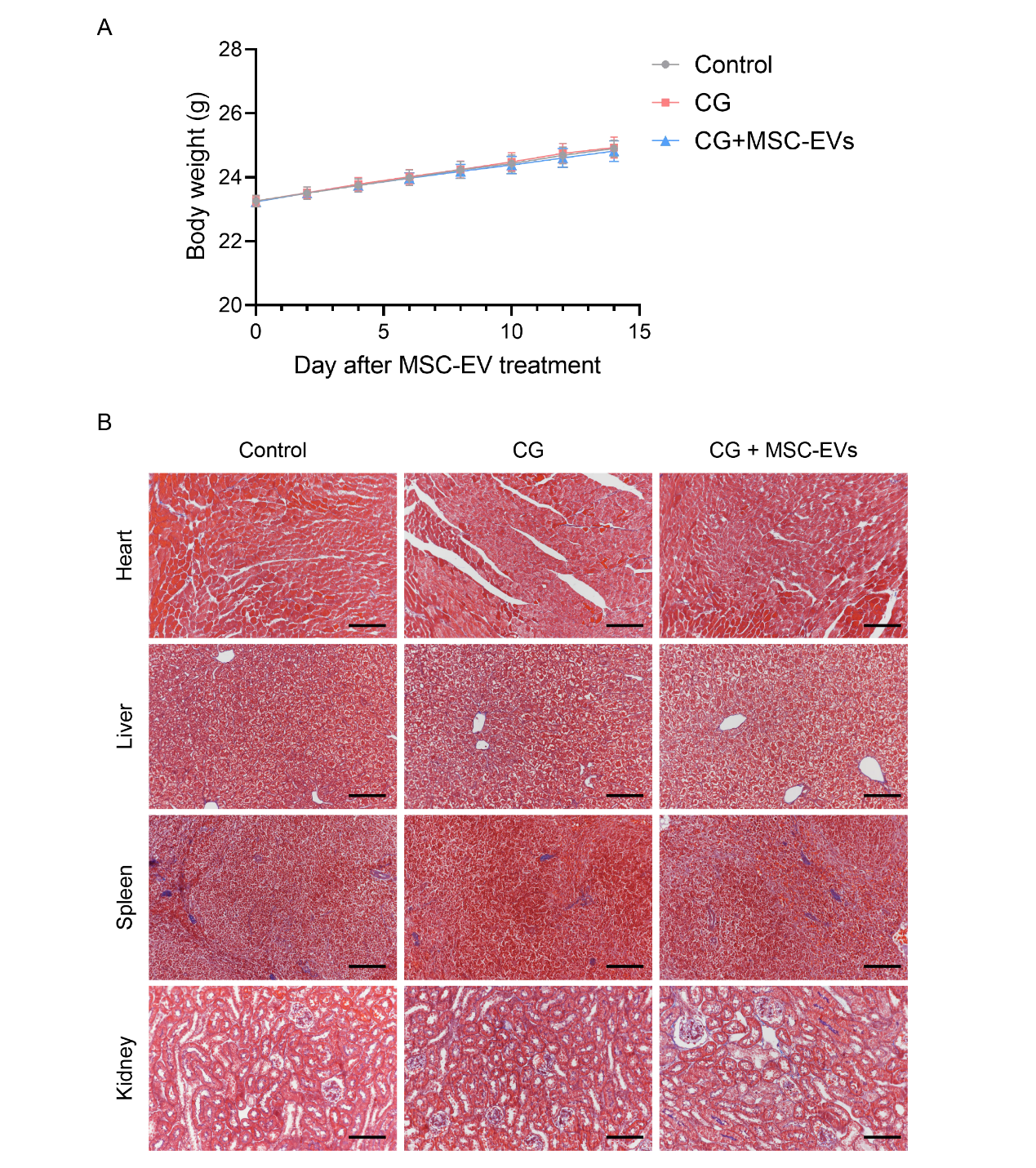


**Figure S1. Safety assessment of MSC-EVs *in vivo*.** (A) The body weight of mice in the Control, CG, and CG + MSC-EVs groups (n=7). (B) Representative images of Masson’s trichrome staining of major organs (heart, liver, spleen, and kidney) in mice from the three groups (n=7). Scale bars, 100 μm.


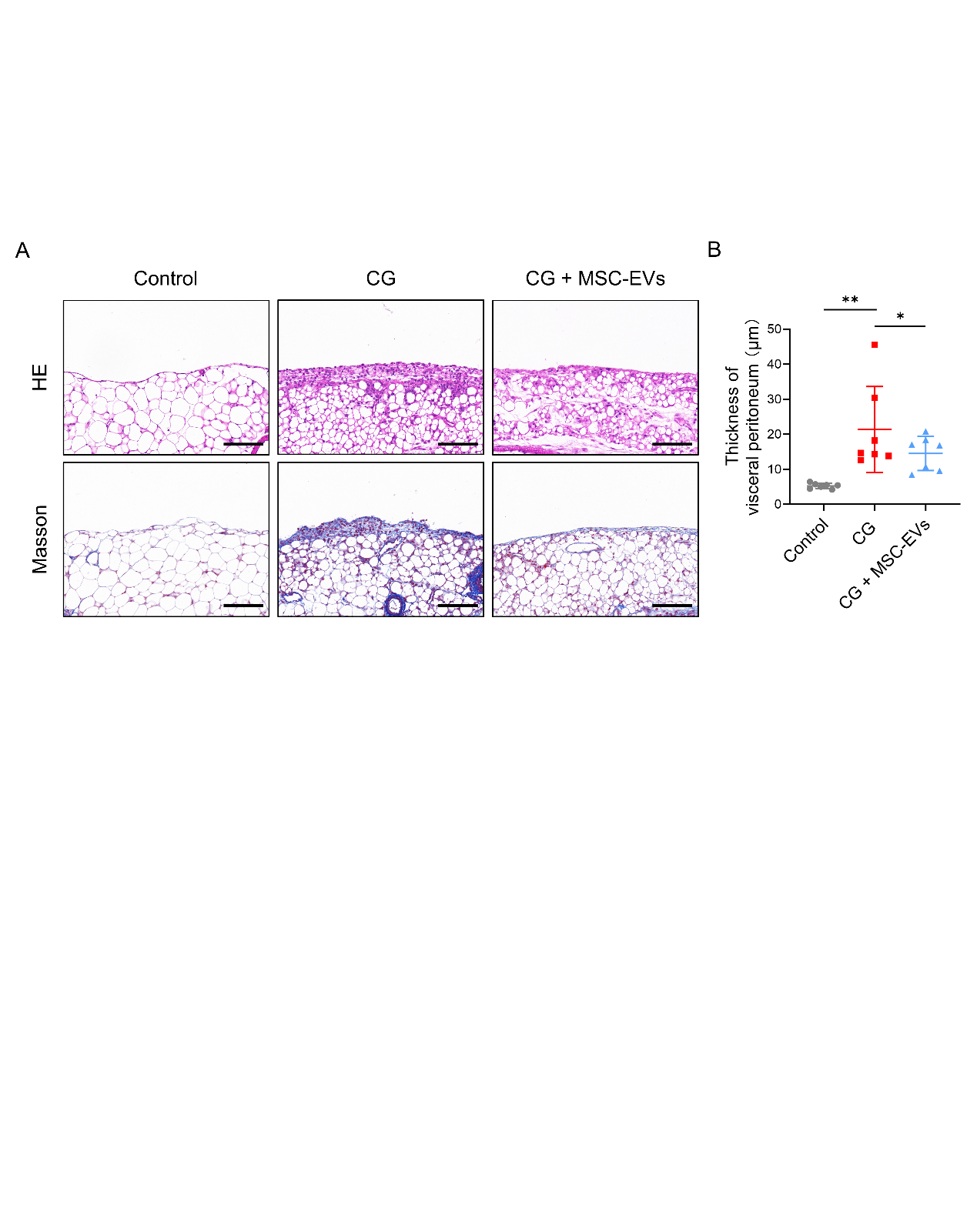


**Figure S2. MSC-EVs inhibit CG-induced peritoneal injury in mice.** (A) Representative H&E staining and Masson’s trichrome staining of visceral peritoneum in the Control, CG, and CG + MSC-EVs groups. Scale bars, 50 μm. (B) Quantitation of the thickness of visceral peritoneum in the three groups (n=7). Data are presented as mean ± SD. **P* < 0.05, and ***P* < 0.01 by one-way ANOVA.


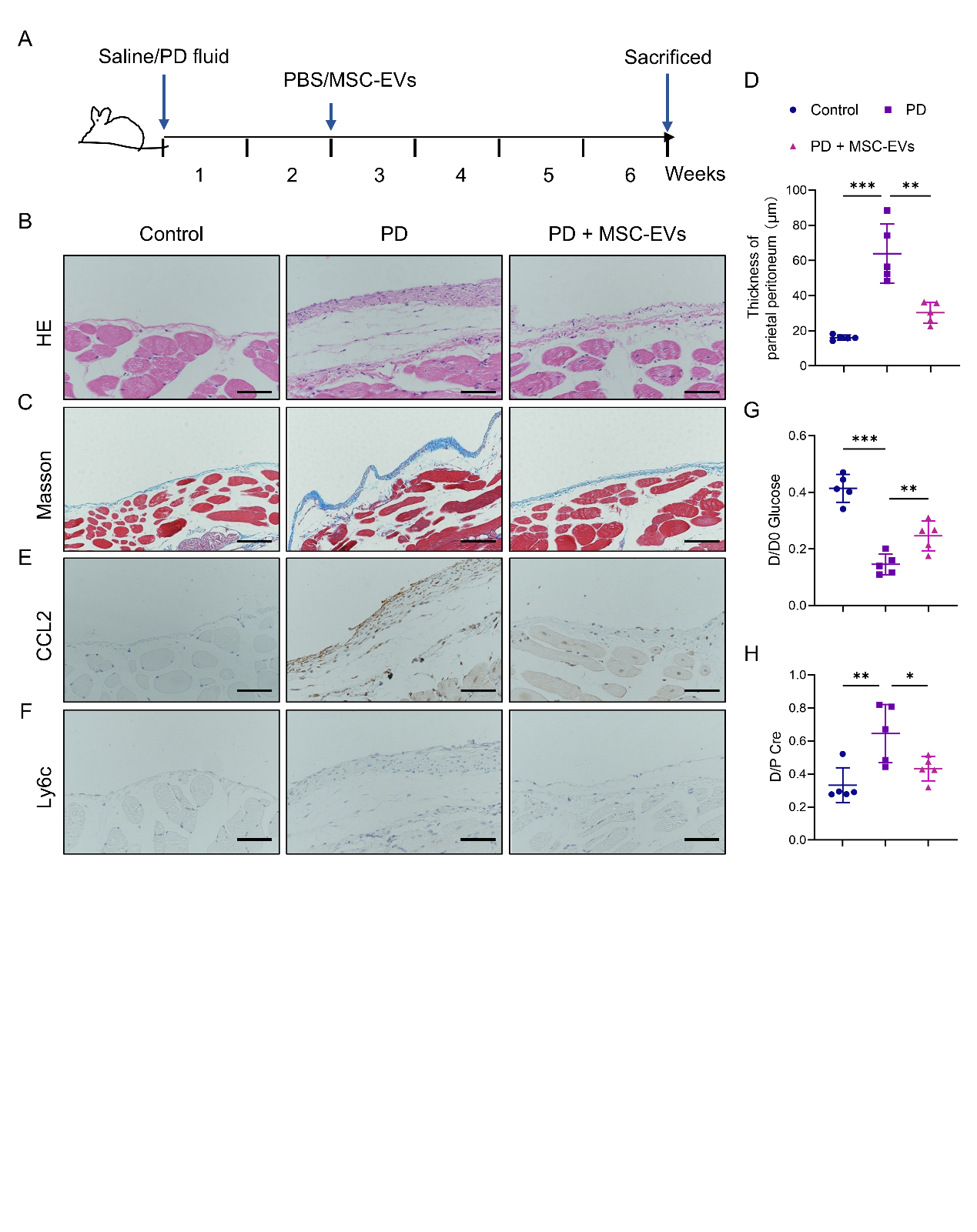


**Figure S3. MSC-EVs suppress PD fluid-induced peritoneal injury in mice.** (A) Overview of the experimental procedure. Mice were given intraperitoneal injections of PD fluid or saline three times weekly for 6 weeks. Starting on day 14, MSC-EVs (5 μg/g body weight) or PBS were administered. (B and C) Representative histology of peritoneal sections from each group: (B) H&E staining; (C) Masson’s trichrome staining (n=5). Scale bars, 100 μm. (D) Measured peritoneal thickness per group (n=5). (E and F) Immunohistochemical staining for CCL2 and Ly6c (n=5). Scale bars, 100 μm. (G and H) Modified peritoneal equilibration test evaluating membrane function: (G) D/D0 glucose; (H) D/P creatinine ratio (n=5). Data are presented as mean ± SD. **P* < 0.05, ***P* < 0.01, ****P* < 0.001 by one-way ANOVA.


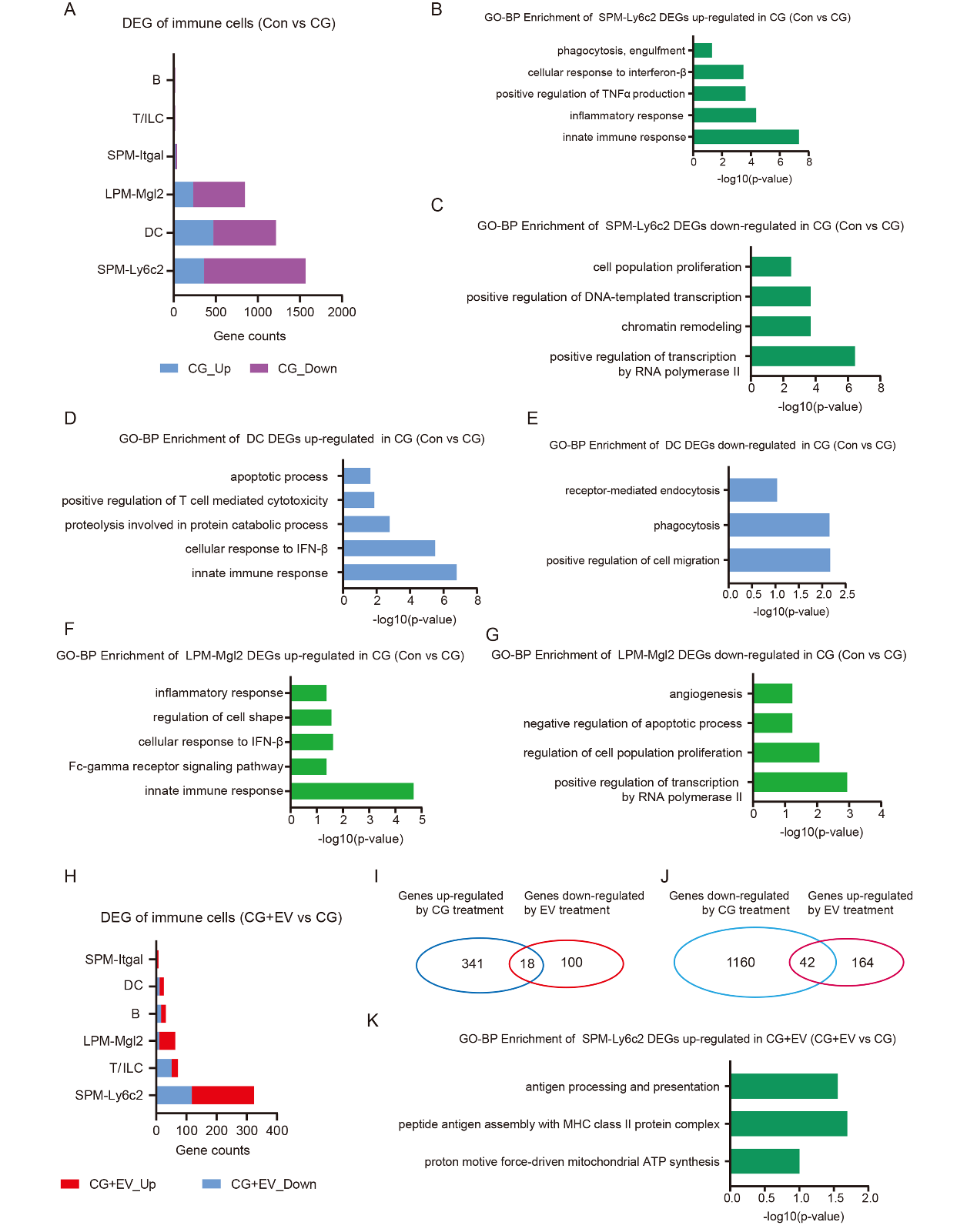


**Figure S4.** **Single-cell transcriptomic profiling analysis of peritoneal immune cells.** (A) The DEG numbers of immune cells between the CON and CG groups. (B-G) GO-BP enrichment analyses of DEGs between CON and CG groups reveal the activation of innate immune and inflammatory response pathways in Ly6c2⁺ SPM (B and C), DC (D and E), and *Mgl2*⁺ LPM (F and G). (H) The DEG numbers of immune cells between the CG and the CG + EV groups. (I) The Venn diagram illustrates the intersection of upregulated genes after CG treatment and downregulated genes after EV treatment. (I) The Venn diagram illustrates the intersection of downregulated genes after CG treatment and upregulated genes after EV treatment. (K) GO-BP enrichment analysis of *Ly6c2*⁺ SPM genes up-regulated in the CG + EV group.


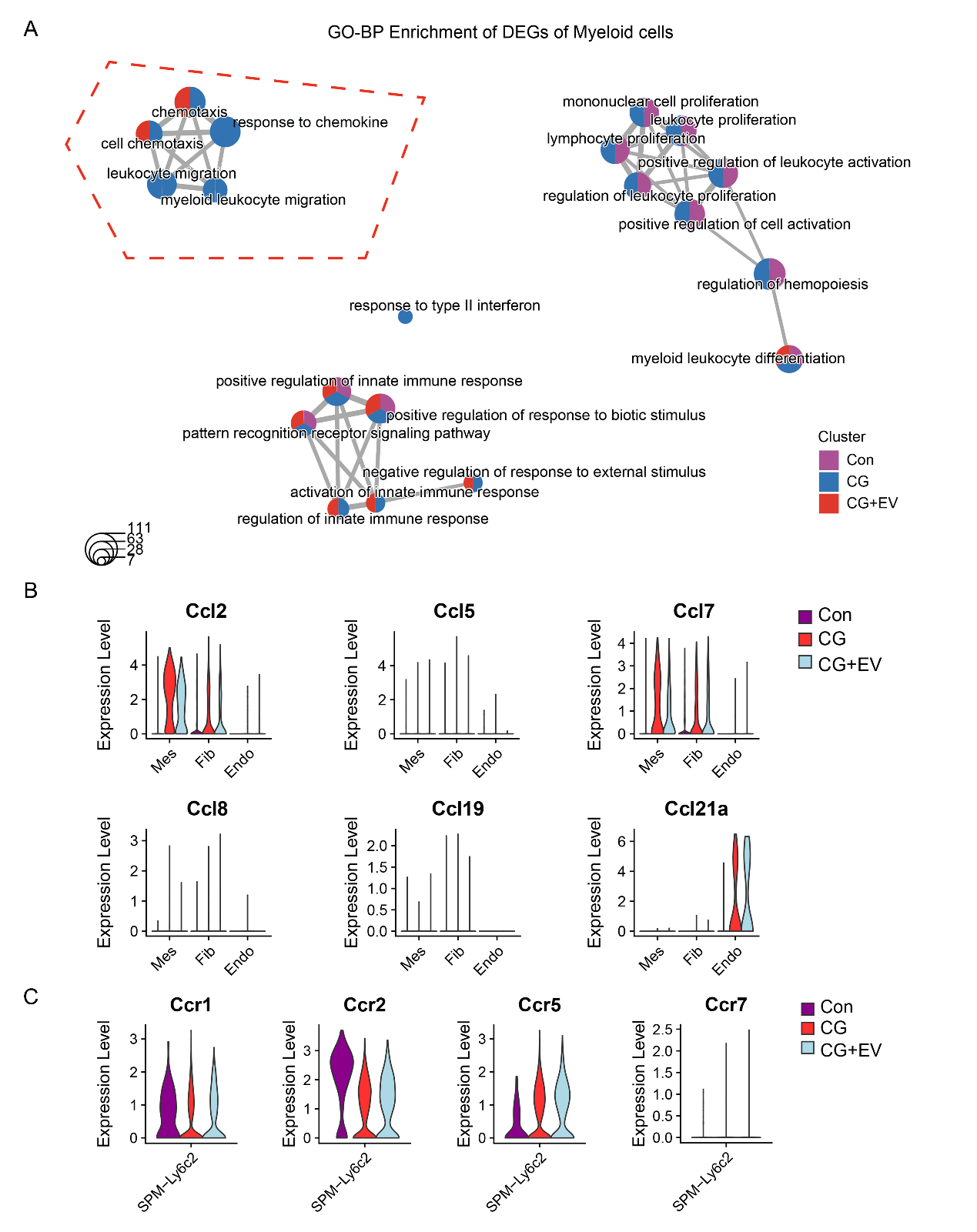


**Figure S5. Single-cell transcriptomic profiling analysis reveals that MSC-EVs significantly reduce the chemotaxis of myeloid cells.** (A) Biological process enrichment analysis of DEGs of myeloid cells among the Con, CG, and CG + EV groups of scRNA-seq. (B) Violin plot showing the expression levels of *Ccl2, Ccl5, Ccl7, Ccl8, Ccl19,* and *Ccl21a* in peritoneal parenchymal cells (mesothelial cells, fibroblasts, and endothelial cells). (C) Violin plot showing the expression levels of *Ccr1, Ccr2, Ccr5*, and *Ccr7* in *Ly6c2*⁺ SPMs.


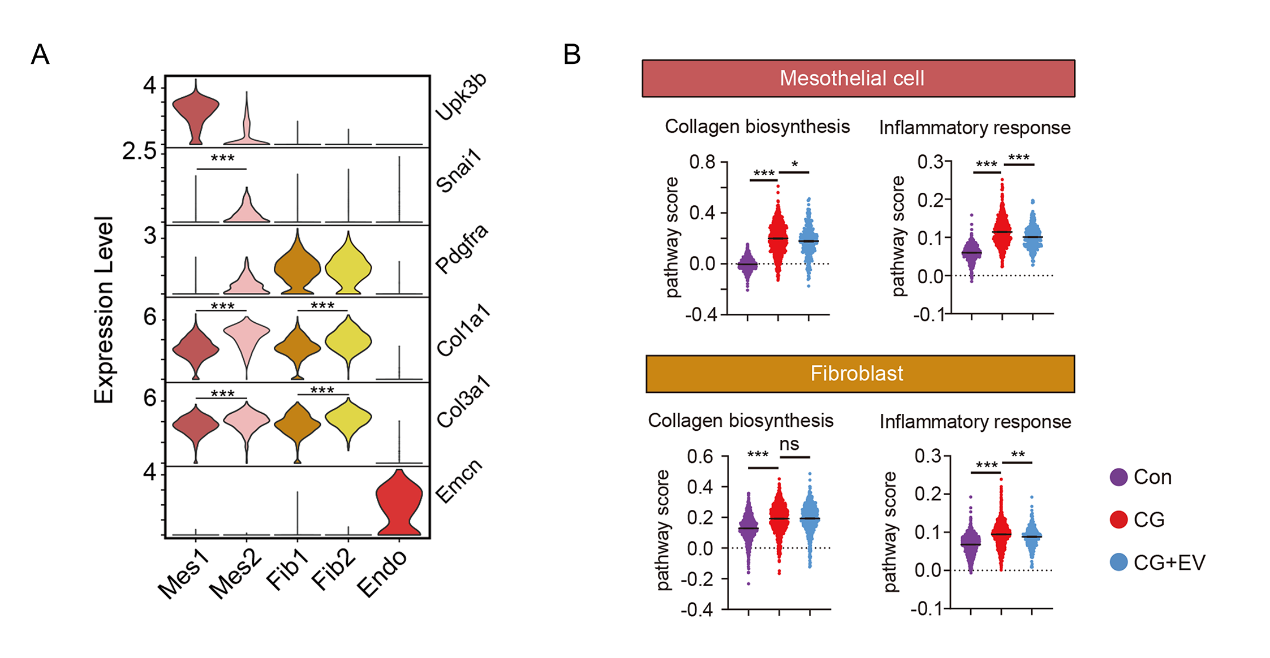


**Figure S6.** **Single-cell transcriptomic profiling analysis of peritoneal parenchymal cells.** (A) Violin plot displaying the peritoneal parenchymal cell subtype annotation markers (*Upk3b, Snai1, Pdgfra, Col1a1, Col1a3*, and *Emcn*). (B) The pathway enrichment scores of collagen biosynthesis and inflammatory response of mesothelial cells and fibroblasts among the Con, CG, and CG + EV groups of scRNA-seq. Data are presented as mean ± SD. **P* < 0.05, ***P* < 0.01, and ****P* < 0.001 by one-way ANOVA. ns: no significance.


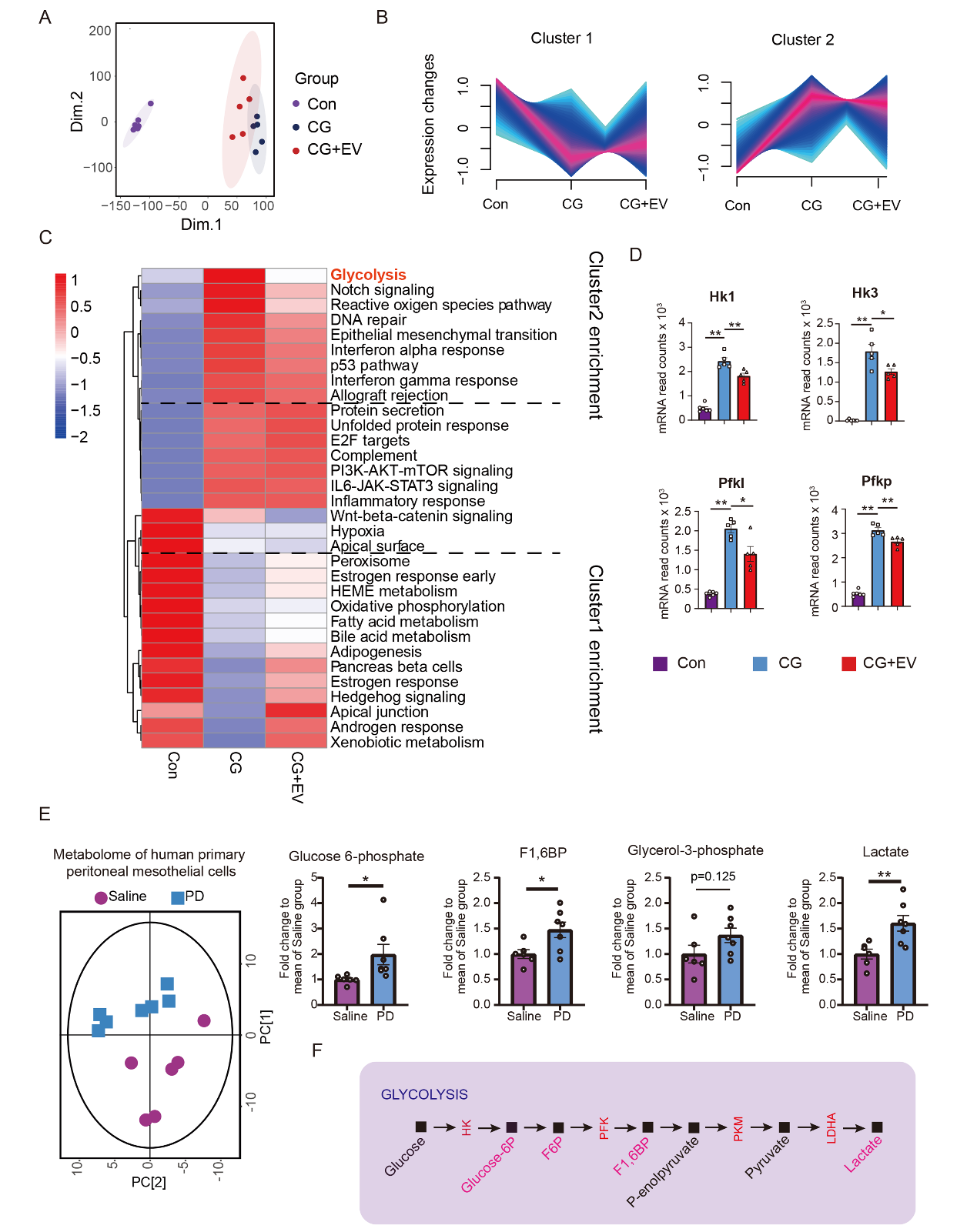


**Figure S7. MSC-EVs suppress the glycolysis of peritoneal tissues induced by CG.** (A) PCA plot of bulk RNA-seq data of mouse peritoneum showing the discrepancies among Con, CG, and CG + EV groups (n=5). (B) Clustering of patterns of relative changes in gene expression among the three groups. (C) The pathway enrichment analysis of the RNA-seq data. (D) The relative mRNA expression of glycolysis-related enzymes (*Hk1, Hk3, Pfkl*, and *Pfkp*) in the three groups. (E) Left: PCA plot of metabolomic profiles of mesothelial cells. Right: the relative expressions of Glucose-6-phosphate, F1,6BP, Glycerol-3-phosphate, and lactate between Saline and PD groups. (F) Changes in key enzymes and metabolites of the glycolytic pathway between the CG and the CG + EV groups. Data are presented as mean ± SD. **P* < 0.05, and ***P* < 0.01 by one-way ANOVA or Student’s t-test.


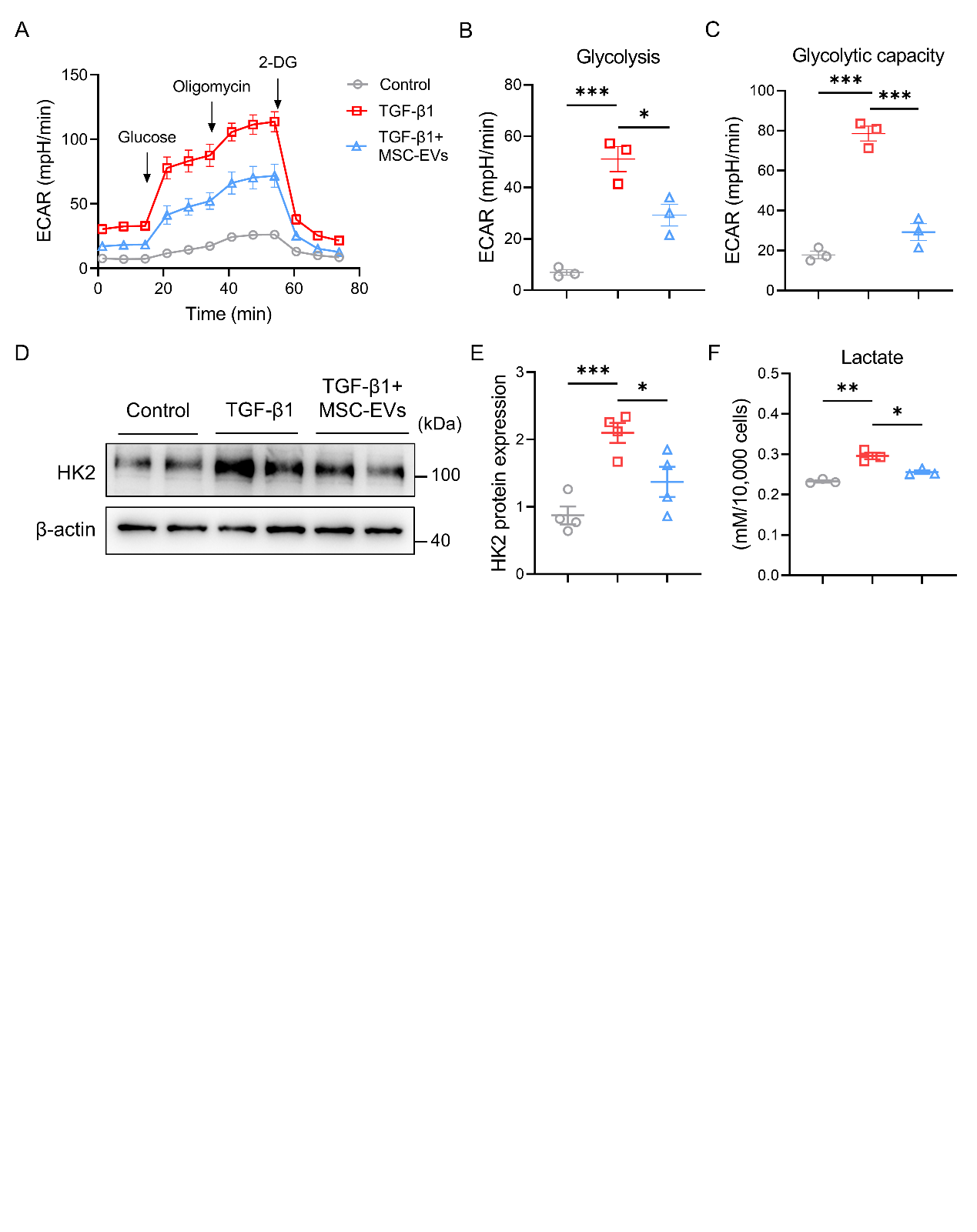


**Figure S8. MSC-EVs inhibit hyperglycolysis and lactate overproduction in injured mesothelial cells.** (A) Measurement of the ECAR in mesothelial cells from the Control, TGF-β1, and TGF-β1 + MSC-EVs groups using a glycolysis stress test. (B and C) Statistical analysis of glycolytic activity in mesothelial cells from the three groups (n=3). (D and E) Representative immunoblotting (D) and quantitative data (E) showing the HK2 protein expressions in mesothelial cells (n=4). (F) The lactate level of mesothelial cell supernatant (n=3). Data are presented as mean ± SD. **P* < 0.05, ***P* < 0.01, and ****P* < 0.001 by one-way ANOVA.


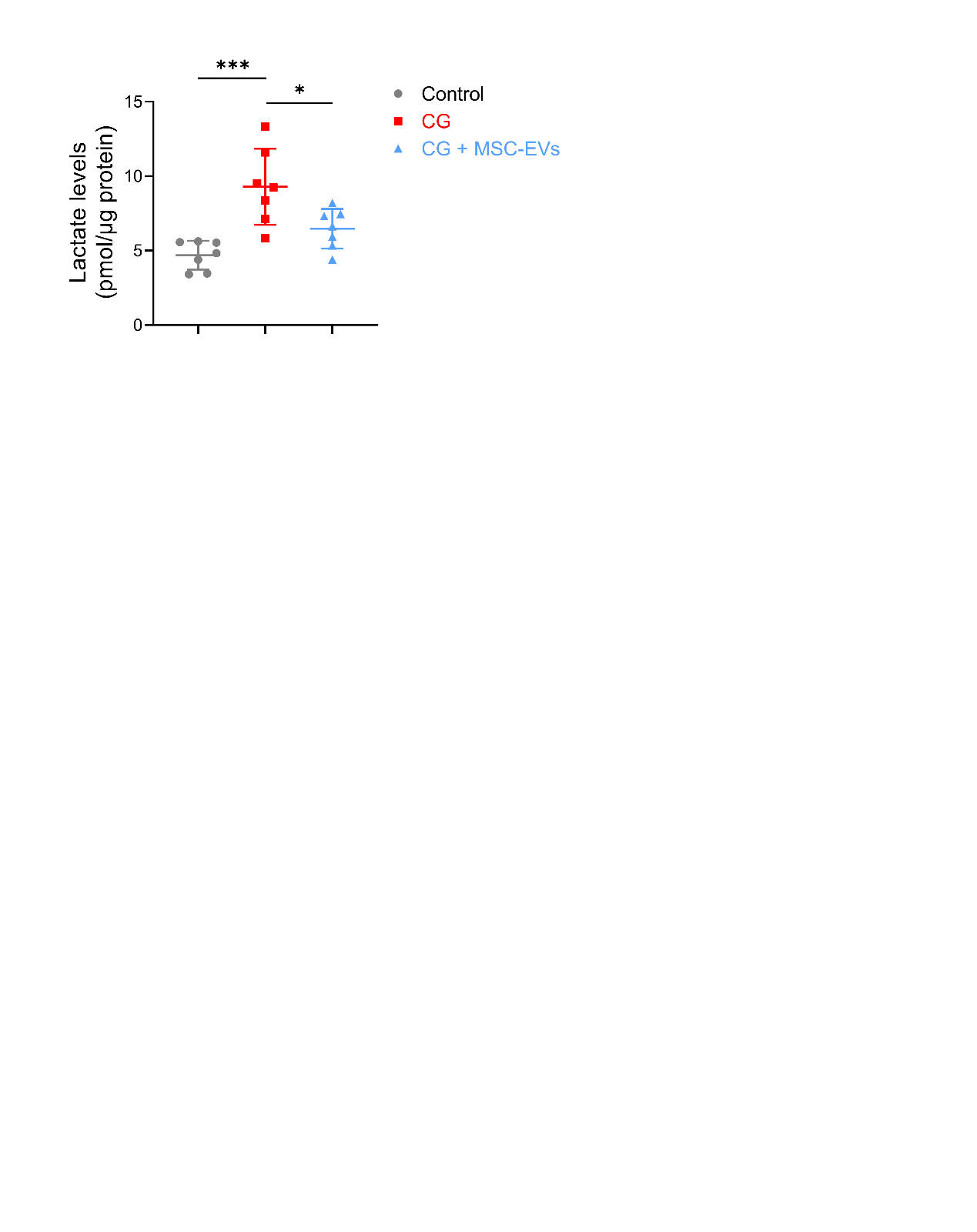


**Figure S9. The lactate level of the mouse peritoneum in the Control, CG, and CG + MSC-EVs groups (n=7).** Data are presented as mean ± SD. **P* < 0.05, and ****P* < 0.001 by one-way ANOVA.


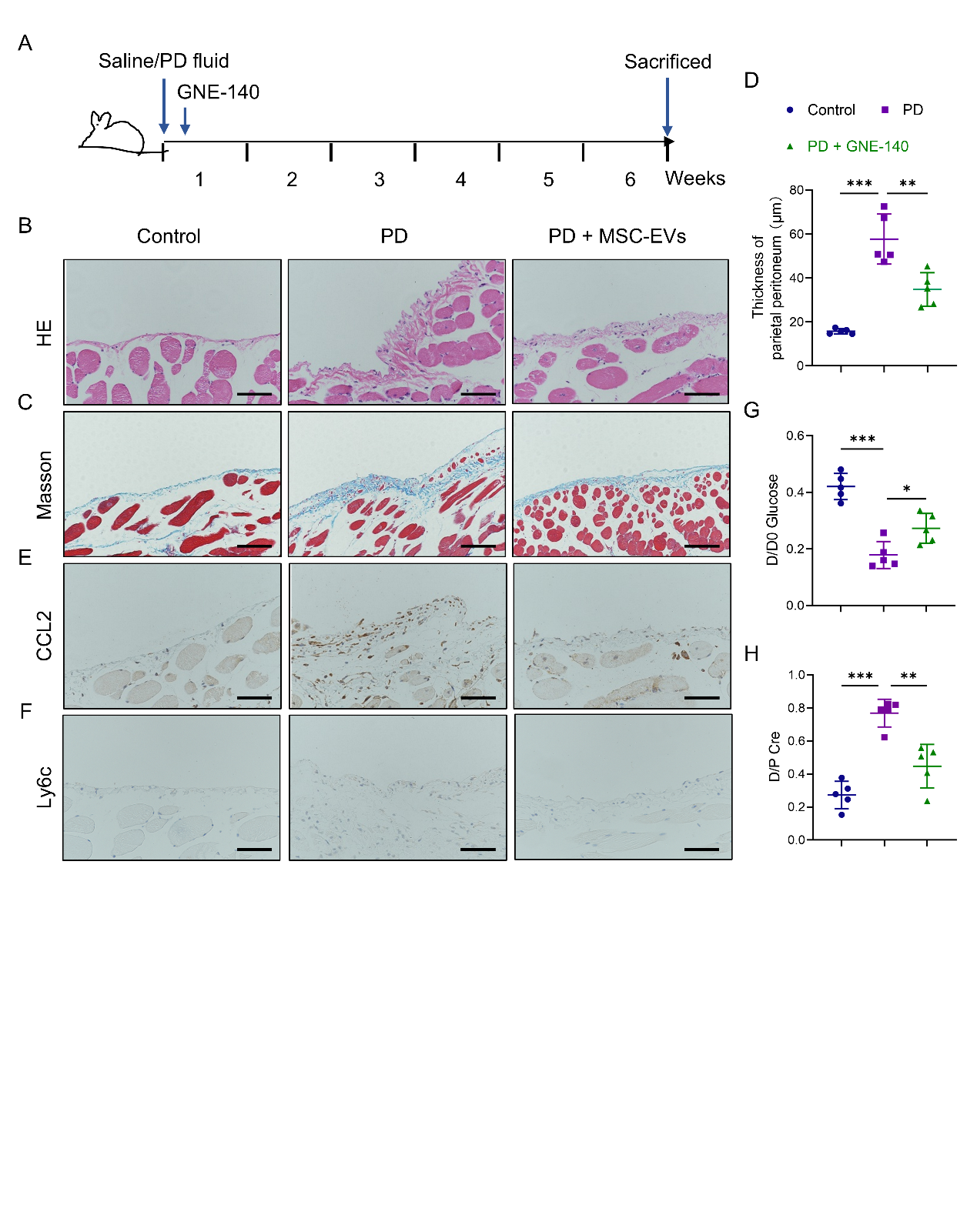


**Figure S10.** **Inhibiting lactate generation reduces inflammatory and fibrotic responses in peritoneal injury induced by PD fluid.** (A) Schematic of the experimental setup. Mice were administered intraperitoneal injections of PD fluid or saline (0.1 mL/g body weight, three times weekly), with or without GNE-140 treatment (5 μg/g body weight, three times weekly), beginning on day 2. (B and C) Representative micrographs of H&E and Masson’s trichrome staining in peritoneal tissues (n=5). Scale bars, 100 μm. (D) Quantified peritoneal thickness values (n=5). (E and F) Representative immunohistochemical staining for CCL2 and Ly6c in peritoneal samples (n=5). (G and H) Modified peritoneal equilibration test assessing membrane transport function: (G) D/D0 glucose ratio; (H) D/P creatinine ratio (n=5 per group). Data are presented as mean ± SD. **P* < 0.05, ***P* < 0.01, ****P* < 0.001 by one-way ANOVA.


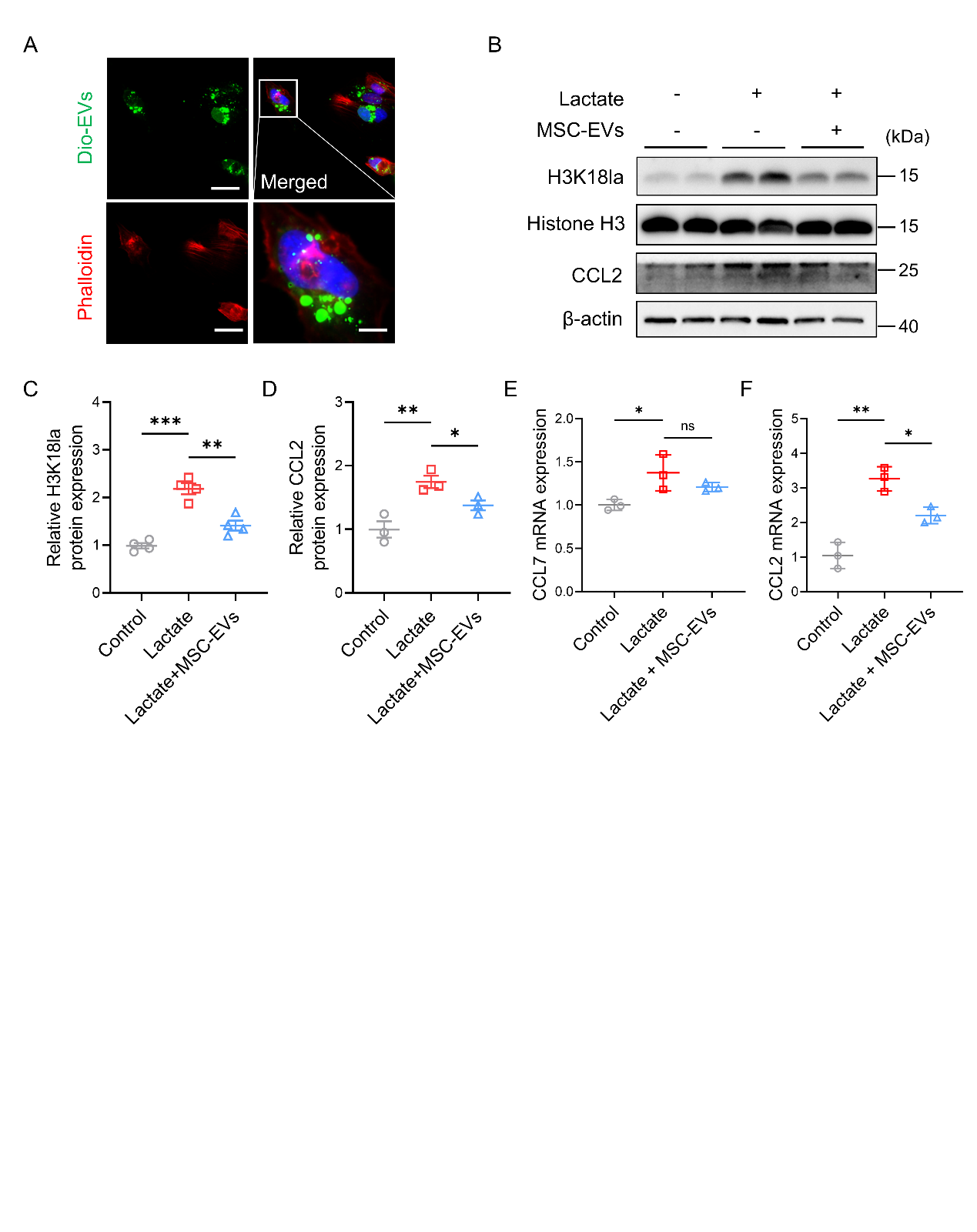


**Figure S11.** **MSC-EVs reduce lactate-induced upregulation of H3K18 lactylation and CCL2 expression in mesothelial cells.** (A) Mesothelial cells were incubated with DiO-labeled MSC-EVs for 24 hours, and representative immunofluorescence images showing the successful delivery of the EVs (green) into Phalloidin-labeled mesothelial cells (red). Scale bars, 100 μm. (B-D) Representative immunoblotting (B) and quantitative data (C and D) showing the protein levels of H3K18la and CCL2 in mesothelial cells treated for 24 hours with 20 mM sodium lactate in the presence or absence of 30 μg/mL MSC-EVs. (E and F) Relative mRNA expression of CCL7 and CCL2 in mesothelial cells across the Control, Lactate, and Lactate + MSC-EVs groups. Data are presented as mean ± SD. **P* < 0.05, ***P* < 0.01, and ****P* < 0.001 by one-way ANOVA. ns: no significance.


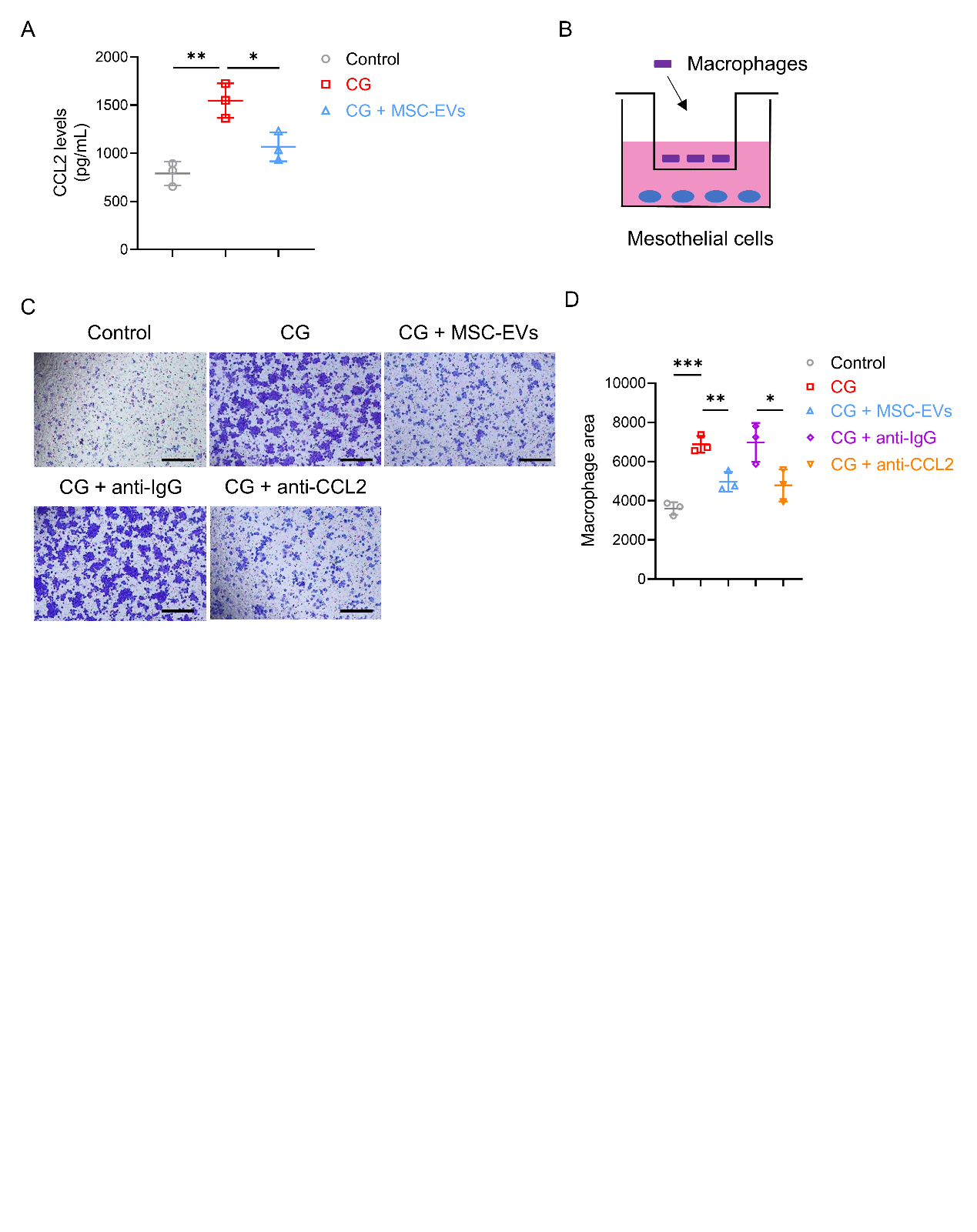


**Figure S12.** **MSC-EVs suppress mesothelial cell-mediated macrophage chemotaxis, potentially through CCL2 downregulation.** (A) The CCL2 levels in conditioned medium from mesothelial cells across the Control, CG, and CG + MSC-EVs groups. (B) Schematic of the indirect co-culture system used for mesothelial cells and macrophages. (C and D) Macrophage migration assays for Control, CG, CG + MSC-EVs, CG + anti-IgG, and CG + anti-CCL2: representative images (C) and quantitative analysis (D). Data are presented as mean ± SD. **P* < 0.05, ***P* < 0.01, and ****P* < 0.001 by one-way ANOVA.


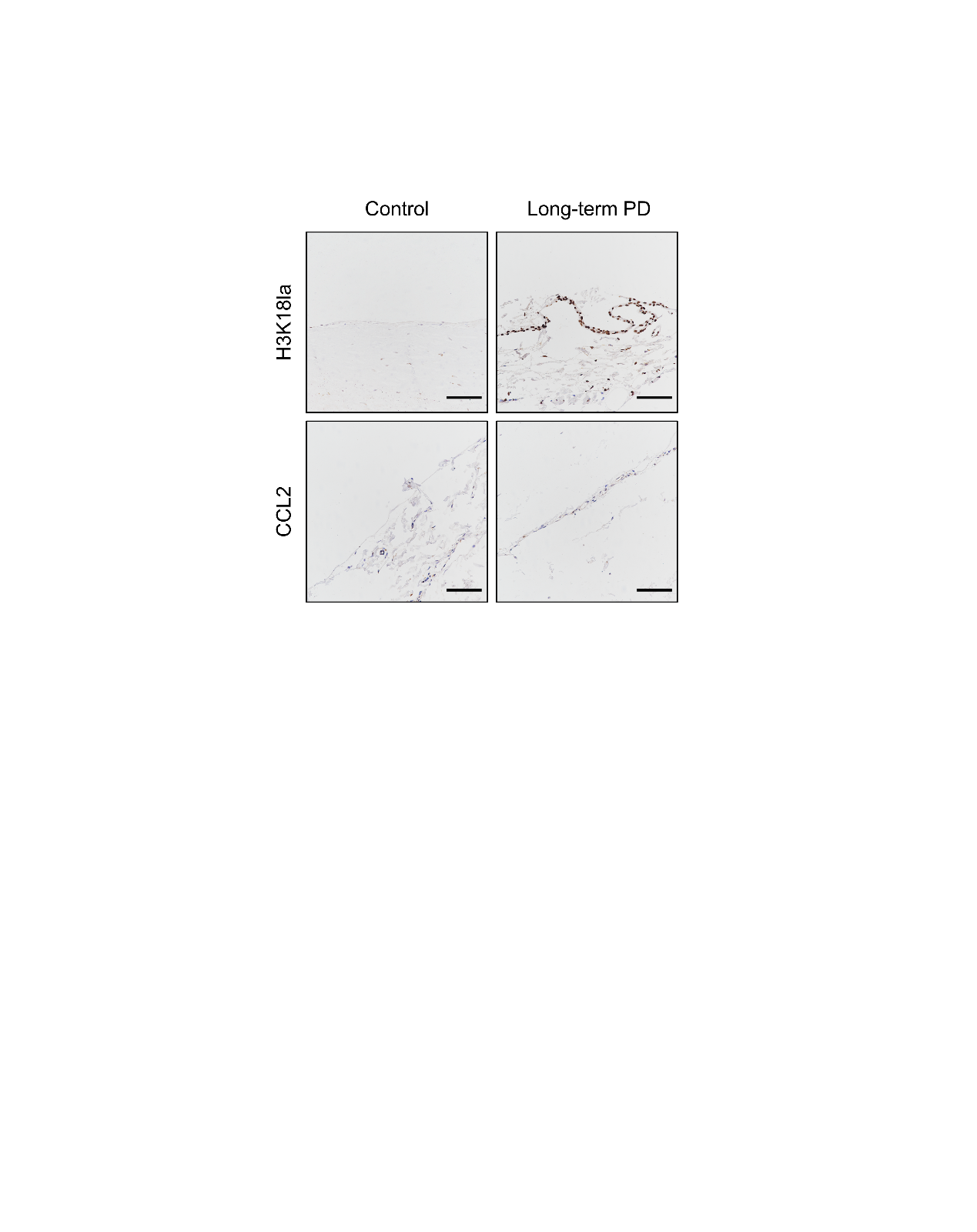


**Figure S13. Representative images of H3K18la and CCL2 staining in human peritoneal tissues from health control and patients undergoing long-term PD.** Scale bars, 100 μm.

**Supplemental Tables**

**Supplemental Table 1. The source and catalog number of antibodies.**

| Product name | Source | catalog number |
| --- | --- | --- |
| FITC-CD105 | Biolegend | 323203 |
| FITC-CD90 | Biolegend | 389803 |
| FITC-CD34 | Biolegend | 343503 |
| FITC-CD45 | Biolegend | 982316 |
| Pan-Kla | PTM Bio | PTM-1401RM |
| H4K8la | PTM Bio | PTM-1415RM |
| H4K12la | PTM Bio | PTM-1411RM |
| H3K9la | PTM Bio | PTM-1419RM |
| H3K18la | PTM Bio | PTM-1427RM |
| HK1 | Proteintech | 19662-1-AP |
| HK2 | Proteintech | 22029-1-AP |
| CD63 | Proteintech | 25682-1-AP |
| TSG101 | Proteintech | 28283-1-AP |
| Calnexin | Proteintech | 10427-2-AP |
| CCL2 | Proteintech | 26161-1-AP |
| Histone H3 | Proteintech | 17168-1-AP |
| β-actin | Proteintech | 66009-1-Ig |
| CD81 | Cell Signaling Technology | 56039S |
| Ly6c | Abcam | ab317272 |
| CCL7 | Abcam | Ab228979 |
| KRT7 | Santa Cruz | sc-23876 |
| α-SMA | Servicebio | GB111364 |
| FN1 | Servicebio | GB114491 |
| CD31 | Servicebio | GB113151 |

**Supplemental Table 2. Primer sequences of real-time qPCR.**

| Gene | Forward | Reverse |
| --- | --- | --- |
| CCL2 | GATCTCAGTGCAGAGGCTCG | TTTGCTTGTCCAGGTGGTCC |
| CCL7 | TGCTCAGCCAGTTGGGATTA | GCTACTGGTGGTCCTTCTGT |
| β-actin | CTGAAGTACCCCATCGAGCA | AGGATGCCTCTCTTGCTCTG |

**Supplemental Table 3. Primer sequence of ChIP-qPCR.**

| Gene | Forward | Reverse |
| --- | --- | --- |
| CCL2 | CCTCACAGAAGGTGGATCTGG | CTGGGCAGACATCAAAAGGGA |
